# Conjugates of urolithin A with NSAIDs, their stability, cytotoxicity, and anti-inflammatory potential

**DOI:** 10.1038/s41598-022-15870-8

**Published:** 2022-07-08

**Authors:** Maciej Korczak, Piotr Roszkowski, Sebastian Granica, Jakub P. Piwowarski

**Affiliations:** 1 Microbiota Lab, Department of Pharmacognosy and Molecular Basis of Phytotherapy, Medical University of Warsaw, ul. Banacha 1, 02-097 Warsaw, Poland; 2 Laboratory of Natural Products Chemistry, Faculty of Chemistry, University of Warsaw, Warsaw, Poland

**Keywords:** Drug development, Drug discovery and development, Lead optimization

## Abstract

Urolithin A (UA, **1**), a gut microbiota postbiotic metabolite is attributed to express interesting biological activities indicated by in vitro, in vivo and clinical studies. Due to its strong anti-inflammatory properties it is considered as a promising lead molecule for further drug development, however, its strong phase II metabolism, severely limits its oral application. Therefore, monoesterified UA derivatives with selected NSAIDs: ibuprofen (Mix **3a**/**3b**), mefenamic acid (Mix **4a**/**4b**), diclofenac (Mix **5a**/**5b**) and aspirin (Mix **6a**/**6b**) were designed. Performed array of stability assays indicated Mix **4a**/**4b** as a most suitable candidate for further studies due to its exceptional stability in human plasma. Thus, we evaluated effects of Mix **4a**/**4b** on cell viability as well as the impact on cytokines secretion in THP-1 derived macrophages and compared it to UA. At high concentration (50 µM) Mix **4a**/**4b** expressed a cytotoxic effect, however at concentration of 5 µM it significantly suppressed TNF-α secretion, and significantly increased ani-inflammatory IL-10 secretion at 10 µM without affecting cell viability. This work has led to selection of a novel UA derivatives, which are stable in solutions and in human plasma as well as posess anti-inflammatory activity towards THP-1 macrophages at non-cytotoxic concentrations.

## Introduction

In recent years, the gut microbiota emerged as a potent modulator of human health, while the abruption of its structure, dysbiosis, is linked with a whole spectrum of diseases, starting from gastrointestinal conditions and ending with mood disorders^1^. Additionally, the usage of probiotics, defined as “live microorganisms which when administered in adequate amounts confer a health benefit on the host”, has reached considerable popularity, despite the questionable efficacy in many cases^2,3^. The positive influence on human well-being is not restricted to the living microbes, but the postbiotics and bacterial metabolites can also impact human health. Though the definition of postbiotics is a subject of ongoing discussion, The International Scientific Association of Probiotics and Prebiotics (ISAPP) proposed the interpretation of postbiotics as a “preparation of inanimate microorganisms and/or their components that confers a health benefit on the host”^4^. According to this wording, the purified metabolites should not be considered as postbiotics per se. Nonetheless, these products of bacterial metabolism are continuously studied, given that their biological activity often surpasses maternal compounds. As a perfect example of purified metabolites marked with high biological activity may serve vitamin B_12_, equol or 8-prenylnaringenin^5,6^.

**Figure 1:**
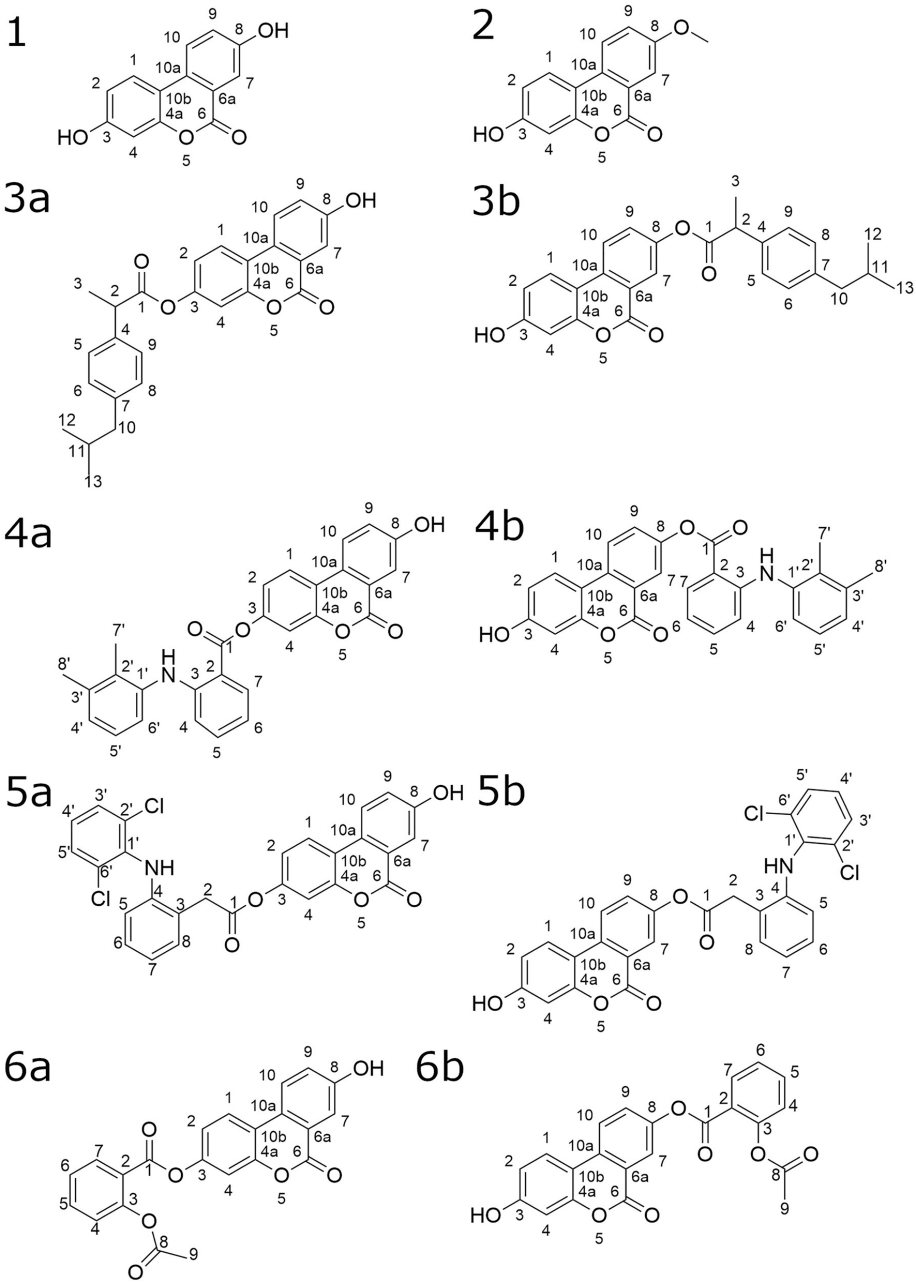
Chemical structure of UA (**1**) and synthesised UADs.

In recent years the gut microbiome-derived metabolites of ellagitannins (ETs) gained a significant attention as candidates for new bioactive molecules^7^. Plants containing high amounts of ellagitannins have been used in traditional medicine for centuries^8–10^. Ellagitannins exhibit several beneficial biological effects in vitro; however, their potential therapeutical use is limited by their low bioavailability. In vivo, after consuming products rich in ellagitannins, they are hydrolysed into hexahydroxydiphenoic acid, which undergoes lactonisation, resulting in the formation of ellagic acid (EA). EA is furtherly metabolised by gut microbiota to urolithins, characterised, contrary to ETs and EA, with good bioavailability^11,12^. Nonetheless, it should be noted that based on the ability to produce urolithins, 3 separate metabotypes can be distinguished in population: metabotype A producing as a final product only urolithin A (UA, compound **1**, Fig. 1), metabotype B producing in addition isourolithin A and urolithin B, and metabotype 0 deprived of appropriate bacteria capable to produce urolithins^13^. Knowing that the administration of ETs does not result in the formation of UA in around 10% of the population (metabotype 0 non-urolithin producers) and UA producing bacterial species are not known yet, the supplementation of ET rich products does not benefit the large group of people^14,15^. The application of UA has been widely studied, for instance, in the context of age-related conditions, muscle function, cancer or inflammation and its health-promoting and anti-inflammatory mechanism of action include induction of mitophagy, suppression of NF-κB signaling pathway and activation of aryl hydrocarbon receptor (AhR) signalling^16^.

Despite unambiguously confirmed pharmocological properties of UA, the pharmakokinetic studies revealed significant limitations in its utilisation as an active dietary supplement or active pharmaceutical ingredient for oral use^7^. The phase 2 metabolism occurring in the intestinal wall and the formation of glucuronide and sulfate conjugates significantly narrows the biological activity of maternal compound^17^. In consequence following the ingestion of either ellagitannins or pure UA they are present in bloodstream and tissues in a conjugated form. These limitations highlight the need for alternative strategies to obtain a pharmacologically sufficient concentrations of UA or its biologically active derivatives in human plasma.

Taking the key role of inflammation in the pathogenesis of various chronic diseases and dubious safety profile of currently used medications, there is a special need for novel, safe anti-inflammatory drugs^18,19^. The modification of the chemical structure of natural products is a well-known strategy in novel drugs development. It was presented that 28% of new drugs approved by the US Food and Drug Administration between 1990 and 2008 originated from naturally occurring substances^20^.

Researchers investigating the anti-inflammatory properties of naturally occurring compounds can use various in vitro models of immune modulation. THP-1 human leukemia monocytic cells that can be differentiated into THP-1 derived macrophages are commonly used as an in vitro inflammatory response model. THP-1 cells present several benefits comparing to human peripheral blood mononuclear cells (PBMC), with high speed of growth or homogeneous genetic background. On the other hand, small differences with PBMC within the expression of receptors or degree of inflammatory response were observed. Nonetheless, THP-1 monocytes and macrophages are considered as suitable for preclinical studies or screening purposes^21^.

Herein we proposed a synthesis of urolithin a derivatives (UADs), with an esterified phenolic hydroxyl group in 3- or 8-position with non-steroidal anti-inflammatory drugs (NSAIDs). Additionally, we performed stability assays of monoesterified mixes of UADs, and for the most stable one tested cytotoxicity and evaluated impact on TNF-α secretion in THP-1 derived macrophages.

## Results and discussion

### Synthesis of UADs

**Figure 2:**
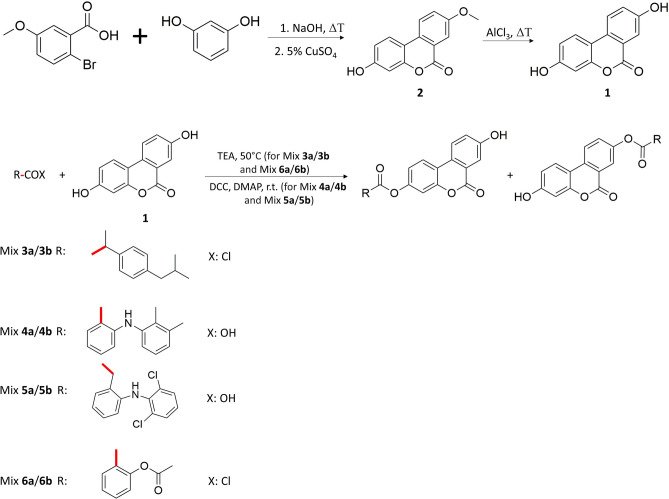
Synthetic strategy used to obtain Mix **3a**/**3b**, Mix **4a**/**4b**, Mix **5a**/**5b** and Mix **6a**/**6b**. Red lines indicate the chemical bonds to the carbonyl group of NSAIDs-based compounds (R-COX) and 5- and 8-substituted UADs.

UA undergoes strong phase II metabolism, being conjugated to glucuronic acid by uridine 5′-diphospho-glucuronosyltransferases (UGTs) enzymes. Knowing that some of the NSAIDs present inhibitory activity towards UGTs, we decided to perform synthesis of esterified UA with ibuprofen, mefenamic acid, diclofenac or acetylsalicylic acid (aspirin)^22,23^. Compounds were chosen as representatives of diverse classes of NSAIDs (propionic acid derivatives, fenamates, acetic acid derivatives and salicylates) and marked with potential different isozyme-specific inhibitory activity toward UGTs^24^. So far there were no studies conducted on synthesis of monoesterified derivatives of UA. Due to the presence of two phenolic groups in UA molecule, initially attempts to conduct esterification resulting in selective obtaining 3-substituted isomer were undertaken. Three-stage strategy was applied to synthesise monoesterified UADs. Firstly, **2** was obtained, and in the next step of synthesis underwent esterification with a particular NSAID. The esterification process was followed by the demethylation of UAD deprived of unblocked –OH groups using AlCl_3_. Unfortunately, during demethylation, the cleavage of ester bound occurred, resulting in the formation of UA. Regardless of lowering the temperature of a chemical reaction or changing the demethylating agent on BBr_3_, the hydrolysis of ester bond (combined with demethylation of UADs in 8-position) repeated. Hence it was decided to synthesise mixtures of monoesterified UADs, with esterified –OH group in 3- or 8-position (Fig. 1). Mixtures of esters with ibuprofen, mefenamic acid, diclofenac and aspirin are referred to as Mix **3a**/**3b**, Mix **4a**/**4b**, Mix **5a**/**5b** and Mix **6a**/**6b**, respectively. In the case of synthesis of ibuprofen and aspirin esters, appropriate acyl chlorides were used, prepared previously according to the literature^25,26^. Mefenamic acid and diclofenac esters were synthesised using DCC/DMAP as a coupling system (Fig. 2).

Hitherto, only the synthesis of etheric derivatives of urolithins was performed^27^. Additionally, obtained compounds were deprived of free hydroxyl groups. In contrast to that study, the goal of our research was to synthesise monosubstituted, esterified UADs. The regioselective synthesis and investigation of monoesterified UADs will provide more detailed information regarding the structure–activity relationship and may benefit further searching of postbiotic metabolites-derived drugs.

### Characterisation of UADs

After successfully synthesising UADs’ mixtures, the identity of obtained compounds was confirmed using the HPLC–MS–DAD method and NMR spectroscopy. All pairs of UADs eluted either as single peaks (Mix **3a**/**3b**, Mix **5a**/**5b**, Mix **6a**/**6b**) or with minimal differences in retention times (Mix **4a**/**4b**) preventing the separation of isomers using C_18_ stationary phases (Fig. S1). The chromatographic strategies to separate the isomeric 3- and 8-substituted monoconjugates were introduced using Phenomenex Kinetex Biphenyl column (150 mm × 2.1 mm, particle size 1.7 μm) and methanol as a mobile phase. No improvement of isomers separation was achieved, what excluded application of preparative HPLC separation using these conditions. However, since the obtained UADs mixtures are similar to the in vivo produced mixture of urolithin A 3- and 8-glucuronides it was decided to conduct further studies on their mixtures. Analogously to our study, the mixture of these phase II metabolites of UA was not separated using the HPLC method^28^. The analysis of mass spectra confirmed the identity of UADs, which gave specific pseudomolecular ions [M-H]^-^ at m/z 415, 450, 505 and 389 for Mix **3a**/**3b**, Mix **4a**/**4b**, Mix **5a**/**5b** and Mix **6a**/**6b**, respectively.

**Figure 3:**
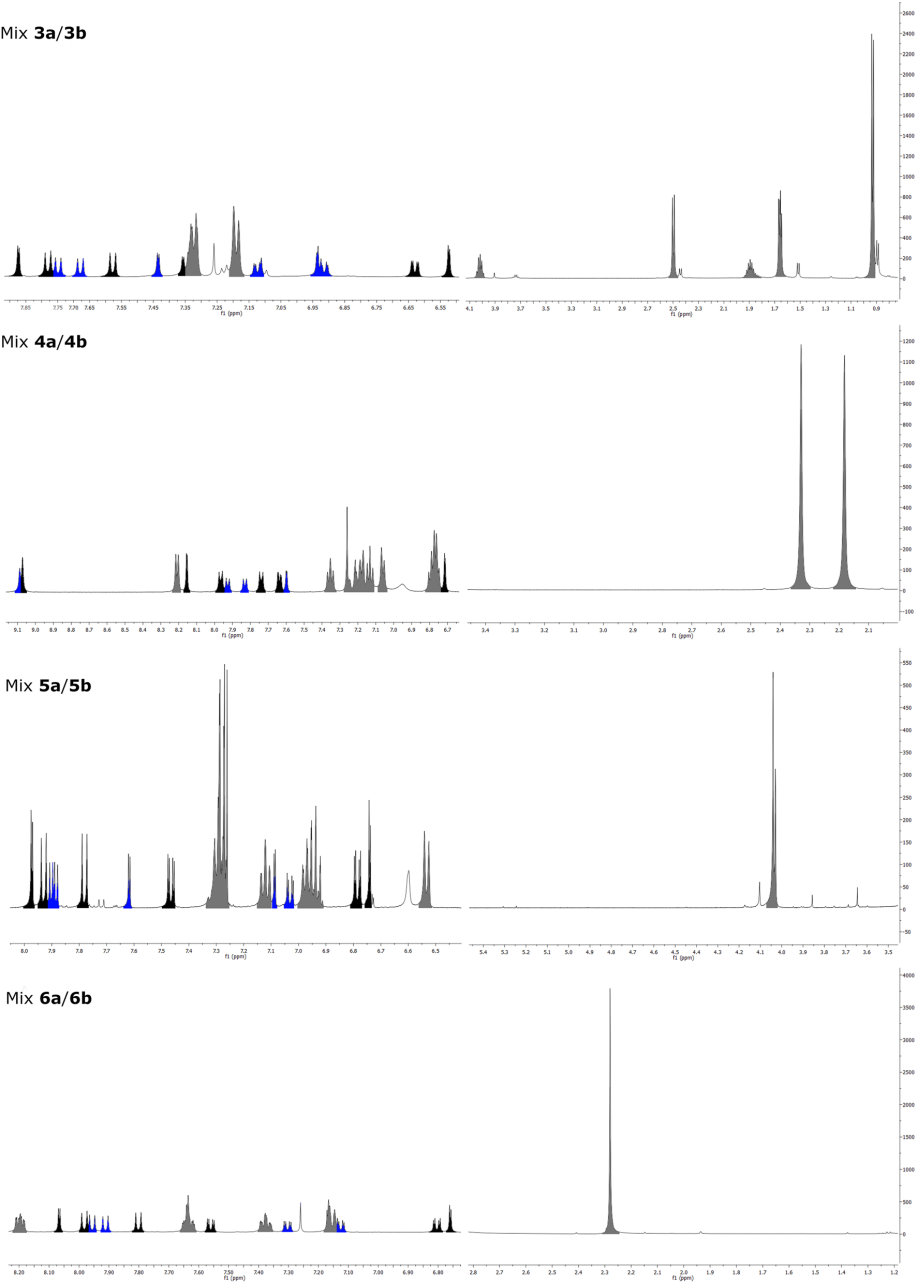
NMR spectra of synthesised UADs. Black: 8-esterified UADs, Blue: 3-esterified UADs, Grey: overlapped signals.

Analogously to previously presented urolithin A glucuronides’ NMR spectra, the downshifts for H2 and H4 of UADs esterified in 3-position and for H7 and H9 of UADs esterified in 8-position were observed (Fig. 3, Tables 1, 2)^28^. Furthermore, a comparison of the proton signals integration ratio revealed the proportion of isomers in mixtures of UADs. The ratio of 3-esterified UADs to 8-esterified UADs were 1:1.27, 1:1.58, 1:1.89 and 1:1.12 for Mix **3a**/**3b**, Mix **4a**/**4b**, Mix **5a**/**5b** and Mix **6a**/**6b**, respectively.

**Table 1:** Mix **3a**/**3b** and Mix **4a**/**4b** spectroscopic data (500 MHz, CDCl_3_).

	**3b**	**3a**	**4b**	**4a**
	δ_H_	δ_H_		
1	7.58 (d, *J* = 8.8 Hz)	7.68 (d, *J* = 8.8 Hz)	7.74 (d, *J* = 9.3 Hz)	7.83 (d, *J* = 9.1 Hz)
2	6.63 (dd, *J* = 8.7, 2.3 Hz)	6.91 (dd, *J* = (8.6, 2.2 Hz)	6.74–6.81 (o)	7.18–7.21 (o)
4	6.52 (d, *J* = 2.3 Hz)	6.94 (d, *J* = 2.0 Hz)	6.71 (d, *J* = 2.1 Hz)	7.21–7.23 (o)
7	7.87 (d, *J* = 2.4 Hz)	7.43 (d, *J* = 2.5 Hz)	8.15 (d, *J* = 2.3 Hz)	7.60 (d, *J* = 2,4 Hz)
9	7.33–7.40 (o)	7.12 (dd, *J* = 8.8, 2.6 Hz)	7.64 (dd, *J* = 7.6, 2.1 Hz)	7.24–7.27 (o)
10	7.78 (d, *J* = 8.8 Hz)	7.75 (d, *J* = 8.6 Hz)	7.97 (d, *J* = 9.3 Hz)	7.93 (d, *J* = 8.7 Hz)
Ibu 2	3.98–4.05 (o)	3.98–4.05 (o)	N/A	N/A
Ibu 3	1.62–1.66 (o)	1.62–1.66 (o)	N/A	N/A
Ibu 5	7.30–7.34 (o)	7.30–7.34 (o)	N/A	N/A
Ibu 6	7.17–7.21 (o)	7.17–7.21 (o)	N/A	N/A
Ibu 8	7.17–7.21 (o)	7.17–7.21 (o)	N/A	N/A
Ibu 9	7.30–7.34 (o)	7.30–7.34 (o)	N/A	N/A
Ibu 10	2.48–2.52 (o)	2.48–2.52 (o)	N/A	N/A
Ibu 11	1.81–1.94 (o)	1.81–1.94 (o)	N/A	N/A
Ibu 12	0.91–0.95 (o)	0.91–0.95 (o)	N/A	N/A
Ibu 13	0.91–0.95 (o)	0.91–0.95 (o)	N/A	N/A
Mef 4	N/A	N/A	6.74–6.81 (o)	6.74–6.81 (o)
Mef 5	N/A	N/A	7.33–7.38 (o)	7.33–7.38 (o)
Mef 6	N/A	N/A	6.74–6.81 (o)	6.74–6.81 (o)
Mef 7	N/A	N/A	8.19–8.23 (o)	8.19–8.23 (o)
Mef 4′	N/A	N/A	7.04–7.08 (o)	7.04–7.08 (o)
Mef 5′	N/A	N/A	7.11–7.16 (o)	7.11–7.16 (o)
Mef 6′	N/A	N/A	7.16–7.20 (o)	7.16–7.20 (o)
Mef 7′	N/A	N/A	2.18 (o)	2.18 (o)
Mef 8′	N/A	N/A	2.33 (o)	2.33 (o)
Mef-NH-	N/A	N/A	9.07 (s)	9.09 (s)

Ibu, Atoms of ibuprofen subunit; Mef, Atoms of mefenamic acid subunit; d, Doublet; dd, Doublet of doublets; o, Overlapped signal.

**Table 2:** Mix **5a**/**5b** and Mix **6a**/**6b** spectroscopic data (500 MHz, CDCl_3_).

	**5b**	**5a**	**6b**	**6a**
	δ_H_	δ_H_		
1	7.78 (d, *J* = 8.8 Hz)	7.89 (d, *J* = 8.8 Hz)	7.80 (d, *J* = 8.8 Hz)	7.91 (d, *J* = 8.8 Hz, 1H)
2	6.79 (dd, *J* = 8.7, 2.4 Hz)	7.03 (dd, *J* = 8.7, 2.3 Hz)	6.80 (dd, *J* = 8.7, 2.4 Hz)	7.13 (dd, *J* = 8.7, 2.3 Hz)
4	6.74 (d, *J* = 2.4 Hz)	7.09 (d, *J* = 2.3 Hz)	6.76 (d, *J* = 2.4 Hz)	7.14–7.18 (o)
7	7.97 (d, *J* = 2.6 Hz)	7.62 (d, *J* = 2.7 Hz)	8.07 (d, *J* = 2.5 Hz)	7.61–7.66 (o)
9	7.46 (dd, *J* = 8.8, 2.6 Hz)	7.26–7.31 (o)	7.56 (dd, *J* = 8.7, 2.5 Hz)	7.30 (dd, *J* = 8.7, 2.7 Hz)
10	7.93 (d, *J* = 8.9 Hz)	7.90 (d, *J* = 8.8 Hz)	7.98 (d, *J* = 8.8 Hz)	7.96 (d, *J* = 8.7 Hz)
Diclo 2	4.02–4.05 (o)	4.02–4.05 (o)	N/A	N/A
Diclo5	7.26–7.31 (o)	7.26–7.31 (o)	N/A	N/A
Diclo 6	6.92–6.99 (o)	6.92–6.99 (o)	N/A	N/A
Diclo 7	6.92–6.99 (o)	6.92–6.99 (o)	N/A	N/A
Diclo 8	6.52–6.55 (o)	6.52–6.55 (o)	N/A	N/A
Diclo 3′	7.26–7.31 (o)	7.26–7.31 (o)	N/A	N/A
Diclo 4′	7.10–7.14 (o)	7.10–7.14 (o)	N/A	N/A
Diclo 5′	7.26–7.31 (o)	7.26–7.31 (o)	N/A	N/A
Asp 4	N/A	N/A	7.14–7.18 (o)	7.14–7.18 (o)
Asp 5	N/A	N/A	7.61–7.66 (o)	7.61–7.66 (o)
Asp 6	N/A	N/A	7.35–7.40 (o)	7.35–7.40 (o)
Asp 7	N/A	N/A	8.18–8.22 (o)	8.18–8.22 (o)
Asp 9	N/A	N/A	2.25–2.30 (o)	2.25–2.30 (o)

Diclo, Atoms of ibuprofen subunit; Asp, Atoms of mefenamic acid subunit; d, Doublet; dd, Doublet of doublets; o, Overlapped signal.

### Stability assays

The high susceptibility to degradation of novel lead compounds may negatively affect the potential usability of promising APIs (Active Pharmaceutical Ingredients). Furthermore, the evaluation of compounds’ stability is often not included during in vitro assays. Therefore, the stability assays for Mix **3a**/**3b**, Mix **4a**/**4b**, Mix **5a**/**5b** and Mix **6a**/**6b**, were performed during our study. Results of stability assays are presented in Table 3. Conditions were selected on the basis of previously described stability-examination protocol and possible exposure of UADs on stress conditions during drug formulation, storage, oral or intravenous administration^29^.

**Table 3:**
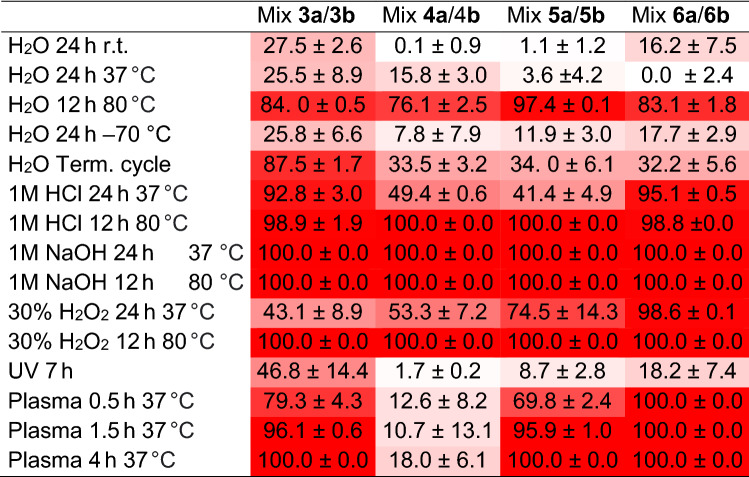
Percentage of degradation of synthesised UADs.

Data express as a mean ± SD.

Despite structural similarities in conjugation mode, synthetised UADs differed substantially in terms of their thermal stability in deionised water. Especially, the highest percentage of degradation either at room temperature or at 37 °C after 24 h incubation was observed for Mix **3a**/**3b**, while around a quarter of the native compound diminished. On the other hand, Mix **5a**/**5b** was highly resistant in those conditions with only 1.1 ± 1.2% and 3.6 ± 4.2% degradation, respectively. After 12 h of incubation in 80 °C, all UADs suffered strong degradation ranging from 76.1 ± 2.5% for Mix **4a**/**4b** to 97.4 ± 0.1% for Mix **5a**/**5b**. In negative temperatures, Mix **3a**/**3b** was the most susceptible to degradation, resulting in almost 26% breakdown of the native compound during incubation in − 70 °C for 24 h and 87.5 ± 1.7% breakdown during the cycle of freezing and thawing. Mix **4a**/**4b** and Mix **5a**/**5b** were characterised with the highest tolerance to incubation in − 70 °C (7.8 ± 7.9% and 11,9 ± 3.0% respectively), while the **Mix 3a**/**3b** presented the highest degradation during the cycle of freezing and thawing − 87.5 ± 1.7%.

UADs were tested for their stability in 1 M HCl, and based on received outcomes, they can be divided into two separate groups. Mix **3a**/**3b** and Mix **6a**/**6b** almost completely vanished after incubation for 24 h in 37 °C or 12 h in 80 °C in an acidic environment. Contrarily, Mix **4a**/**4b** and Mix **5a**/**5b** moderately tolerate 24 h incubation in 37 °C with 1 M HCl (49.4 ± 0.6% and 41.4 ± 4.9% degradation, respectively). Nonetheless, Mix **4a**/**4b** and Mix **5a**/**5b** were also undetected after incubation at a higher temperature for 12 h.

In terms of UADs’ stability in basic conditions, all native compounds diminished completely or almost completely, no matter of time and temperature of incubation in 1 M NaOH.

UADs’ susceptibility to oxidative stress varied widely, and Mix **3a**/**3b** turned out to be the least affected by the incubation in 30% H_2_O_2_ at 37 °C for 24 h with only 43.1 ± 8.9% degradation. At the other end of the scale, the level of Mix **6a**/**6b** was decreased by almost 99% compared to the control. Unsurprisingly, none of UADs’ peaks was detected after incubation at 80 °C for 12 h.

The breakdown of UADs exposed to UV light was tested as well. While Mix **4a**/**4b** was almost unaffected by UV light (1.7 ± 0.2% degradation), Mix **5a**/**5b**, Mix **6a**/**6b**, Mix **3a**/**3b** were partially degraded (8.7 ± 2.8, 18.2 ± 7.4, 46.8 ± 14.4% degradation, respectively).

All UADs except Mix **4a**/**4b** underwent strong degradation in human plasma incubated at 37 °C, resulting in the total absence of UADs’ peaks after 4 h. Surprisingly, Mix **4a**/**4b** was marked with good toleration to the human plasma environment with a ratio of degradation of 18.0 ± 6.1% after 4 h.

### Cytotoxicity

**Figure 4:**
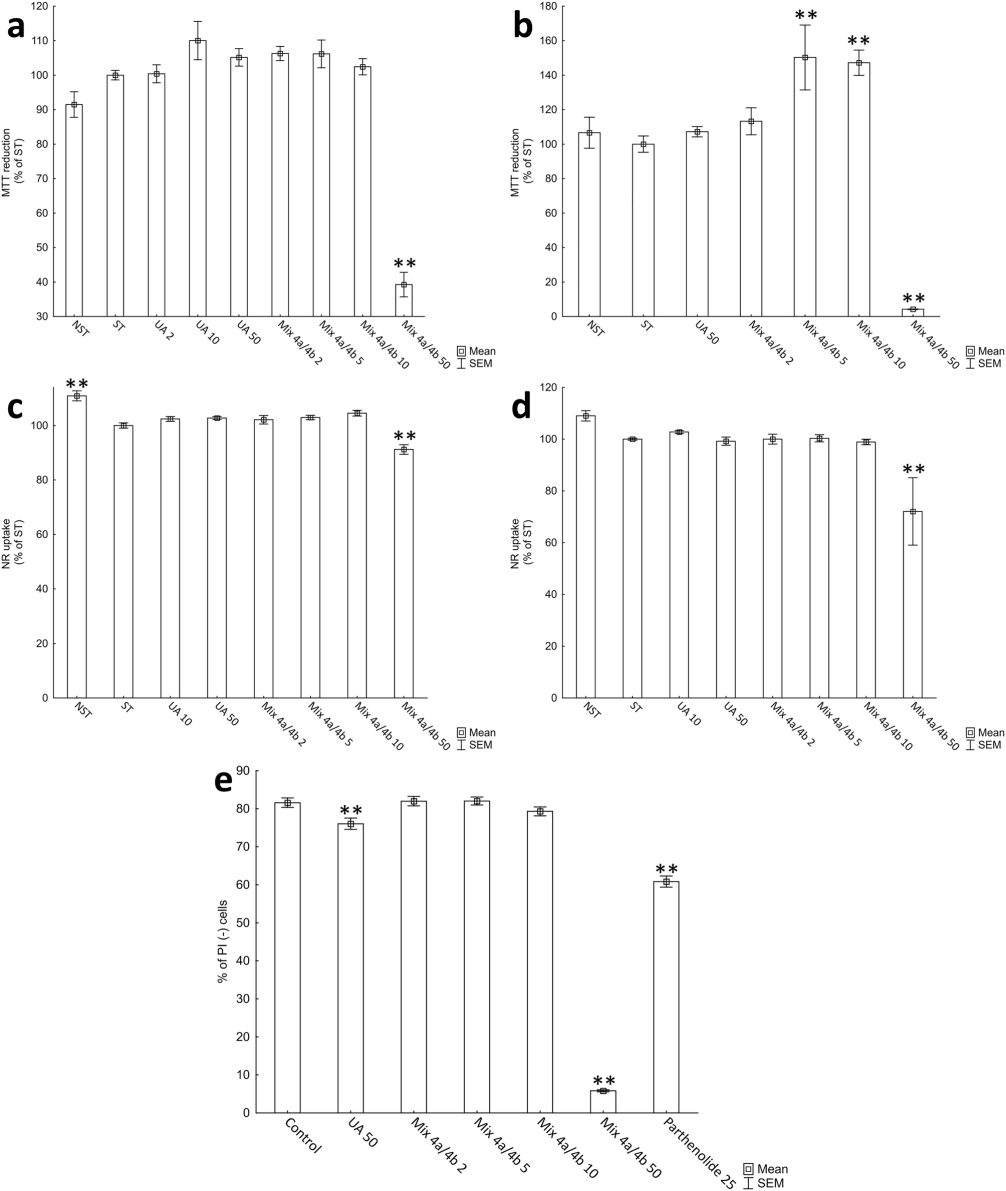
Cytotoxicity of UA and Mix **4a**/**4b**. (**a**) Effects of UA and Mix **4a**/**4b** on THP-1 derived macrophage viability measured using MTT assay after treatment with the test compounds for 1 h and subsequent stimulation with LPS for 3 h. (**b**) Effects of UA and Mix **4a**/**4b** on THP-1 derived macrophage viability measured using MTT assay after treatment with the test compounds for 1 h and subsequent stimulation with LPS for 24 h. (**c**) Effects of UA and Mix **4a**/**4b** on THP-1 derived macrophage viability measured using NRU assay after treatment with the test compounds for 1 h and subsequent stimulation with LPS for 3 h. (**d**) Effects of UA and Mix **4a**/**4b** on THP-1 derived macrophage viability measured using NRU assay after treatment with the test compounds for 1 h and subsequent stimulation with LPS for 24 h (**e**) Effects of UA and Mix **4a**/**4b** on THP-1 monocyte viability using PI flow cytometric assay. Data expressed as a mean ± SEM. Numbers next graph labels indicate tested concentration (in µM). Statistical comparisons were made using the parametric method of one-way ANOVA, followed by the Dunnett's post hoc test. Statistical significance: **p < 0.01 versus ST group or Control in PI flow cytometric assay. *NST* Non-stimulated control, *ST* LPS stimulated control.

Taking into account the exceptional stability of Mix **4a**/**4b** in human plasma, this derivatives were chosen for further in vitro assays. The cytotoxicity of Mix **4a**/**4b** on THP-1 derived macrophages was tested using a standard MTT assay performed after administration of UADs, one hour resting and stimulation of cells with LPS for 3 or 24 h. Mix **4a**/**4b** did not exhibit cytotoxic effect up to 10 µM (Fig. 4a,b, Tables S1, S2). However, when 50 µM concentration was used, cell viability significantly declined. Additionally, the impact of UA on THP-1 macrophages viability was assessed, and no cytotoxic effect was observed in the tested concentration range (2–50 µM), similarly to previously obtained results^8,17^. The paradoxical increased viability after 24 h incubation with 5 and 10 µM Mix **4a**/**4b** may result from the dependance of MTT assay on metabolic rate^30^. Furthermore, the cytotoxic effect of 50 µM Mix **4a**/**4b** was independently proved using Neutral Red Uptake (NRU) assay performed 3 and 24 h after LPS stimulation (Fig. 4c,d, Tables S3, S4).

Parallelly, the cytotoxicity of UA and Mix **4a**/**4b** on THP-1 monocytes was investigated using flow cytometry analysis of PI-stained cells with 25 µM parthenolide as a positive control (Fig. 4e, Table S5). As previously described, 25 µM parthenolide decreased THP-1 viability^31^. Similarly to results obtained for THP-1 derived macrophages using MTT and NRU assays, Mix **4a**/**4b** did not influence THP-1 monocytes viability up 10 µM. The drastic drop in cell viability was observed after 24-h incubation with 50 µM Mix **4a**/**4b.** Slight, yet statistically significant decline in monocytes viability after administration of 50 µM UA was observed.

### Impact of Mix 4a/4b on cytokines secretion

**Figure 5:**
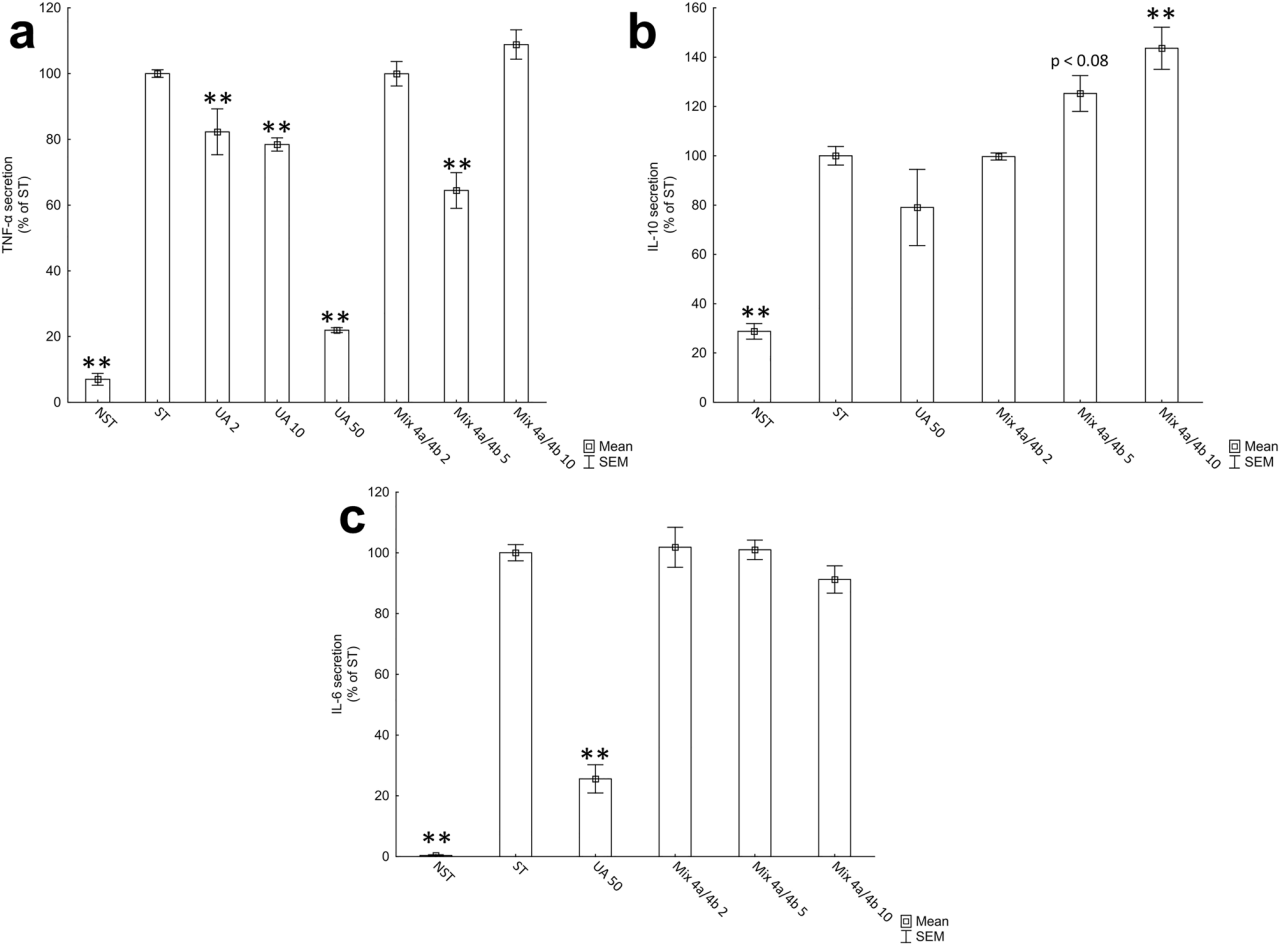
Evaluation of anti-inflammatory activity of UA and Mix **4a**/**4b** using LPS stimulated THP-1 derived macrophages. (**a**) Effects of UA and Mix **4a**/**4b** on TNF-α secretion after treatment with the test compounds for 1 h and subsequent stimulation with LPS for 3 h. (**b**) Effects of UA and Mix **4a**/**4b** on IL-10 secretion after treatment with the test compounds for 1 h and subsequent stimulation with LPS for 24 h. (**c**) Effects of UA and Mix **4a**/**4b** on IL-6 secretion after treatment with the test compounds for 1 h and subsequent stimulation with LPS for 24 h. Data expressed as a mean ± SEM. Numbers next graph labels indicate tested concentration (in µM). Statistical comparisons were made using the parametric method of one-way ANOVA, followed by the Dunnett’s post hoc test. Statistical significance:**p < 0.01 versus ST group. *NST* Non-stimulated control, *ST* LPS stimulated control.

The TNF-α secretion of LPS-stimulated THP-1 derived macrophages was assessed using ELISA kits. UA was chosen as a control compound based on the well-established inhibitory activity of UA on TNF-α secretion and thoroughly examined the underlying intracellular mechanism of action^16^. As expected, UA exhibited an inhibitory effect on TNF-α secretion in a dose-dependent manner (Fig. 5a, Table S6).

Mix **4a**/**4b** affected TNF-α secretion 3 h after stimulation. While the significant inhibitory effect on TNF-α level was observed already at 5 µM Mix **4a**/**4b**, this impact was not detected using concentration of 10 µM (Fig. 5a, Table S6). The lack of concentration-dependency of determined activity between 5 and 10 µM concentrations (not observed in the case of UA), can arise from the hybrydic properties of the Mix **4a**/**4b**. As described above, UA exhibits an inhibitory effect on TNF-α secretion, affecting the NF-κB signalling pathway in cells. On the other hand, mefenamic acid non-selectively inhibits COX-2, and it is known, that inhibition of this enzyme can increase TNF-α expression in THP-1 macrophages^32,33^. The additional set of experiments using THP-1 derived macrophages confirmed the stimulating effect of mefenamic acid on TNF- α secretion (Fig. S7). Thus, the contrary effect of two Mix **4a**/**4b** subunits might explain the dose-independent manner of action and will be closely evaluated in future research. Moreover, the cytotoxic effect of Mix **4a**/**4b** at 50 µM concentration was presented. After administration of 10 µM Mix **4a**/**4b**, we did not observe the decrease of the viability of THP-1 macrophages. Nevertheless, it was previously demonstrated that despite the not observed decline in THP-1 cells viability, the induction of apoptotic intracellular signalling pathways, including caspases activity and parallel upregulated production of proinflammatory TNF-α, were detected after administration of several stressors^34,35^.

Additionally, the impact of UA and Mix **4a**/**4b** on anti-inflammatory interleukin-10 (IL-10) and interleukin-6 (IL-6) secretion 24 h after stimulation with LPS was tested (Fig. 5b,c, Tables S7, S8). In accordance with previously published results, UA did not significantly affect IL-10 secretion^17^. Interestingly, Mix **4a**/**4b** increased levels of IL-10 comparing to stimulated control in a dose-dependent manner, reaching statistical significance at a concentration of 10 µM. Consistent with hitherto presented results, 50 µM UA inhibited secretion of IL-6^36^. Contrary to this specific UA activity, Mix **4a**/**4b** did not affect IL-6 secretion in THP-1 derived macrophages. While the mefenamic acid alone did not influence IL-10 secretion, it increased IL-6 production in a dose-dependent manner (Fig. S7). These findings suggest distinctive mechanism of action of Mix **4a**/**4b** from that previously determined for UA^37^.

## Conclusion

In summary, we performed the successful synthesis of mixtures of UADs monoesterified to ibuprofen (Mix **3a**/**3b**), mefenamic acid (Mix **4a**/**4b**), diclofenac (Mix **5a**/**5b**) and aspirin (Mix **6a**/**6b**). Despite similarities in conjugation mode, obtained compounds fundamentally differ in terms of their stability, and contrary to other UADs, Mix **4a**/**4b** well tolerated the human plasma environment. The determined impact of Mix **4a**/**4b** on TNF-α secretion may result either from their cytotoxic effect at high concentration or the opposing activity of Mix **4a**/**4b** subunits at lower concentrations. The influence of Mix **4a**/**4b** on IL-10 secretion and lack of activity towards IL-6 secretion imply mechanism of action, which is different from that previously determined for UA^37^. Presented results create a novel approach in anti-inflammatory drug development, focusing on chemical modification of the structure of postbiotic metabolites. Additional studies should be oriented on the synthesis of monoesterified UADs and close investigation of their intracellular mechanism of action. Further work evaluating UADs bioavailability in vitro, with the special interest in the role of UGTs, are currently in progress.

## Methods

### Synthetic method

#### Synthesis of 3-hydroxy-8-methoxy-6H-benzo[c]chromen-6-one (2)

The synthesis of the first intermediate, **2**, was performed according to the previously described protocol^38^. Briefly, after heating under reflux mixture of resorcinol (1 g; 9.08 mmol, 2.1 eqv), 2-Bromo-5-methoxybenzoic acid (1 g; 4.33 mmol, 1.0 eqv) and NaOH (0.375 g; 9.38 mmol, 2.2 eqv) in water (4.5 mL) for 30 min, a 5% aqueous solution of CuSO4 (1.8 ml; 0.56 mmol, 0.1 eqv) was added dropwise. Afterwards, the solution was heated for additional 10 min. The obtained precipitate was filtered, dried and the identity of **2** was confirmed using the UHPLC-DAD-MS method; 610 mg (58%).

#### Synthesis of 3,8-dihydroxy-6H-benzo[c]chromen-6-one (UA, 1)

The goal of this procedure was to obtain mixtures of UADs with esterified 3- or 8- position. A solution of **2** (0.51 g; 2.11 mmol, 1 eqv) and AlCl_3_ (1.46 g; 10.95 mmol, 5.2 eqv) in chlorobenzene was refluxed, and the demethylation process was monitored using the UHPLC-DAD-MS method. After complete demethylation of **2**, resulting in the formation of UA, a cooled mixture was added to ice. In the next step, the mixture was extracted with diethyl ether (3 × 125 ml). Subsequently, the organic solvent was evaporated on a rotary evaporator equipped with a vacuum system to reduce the pressure. The identity of obtained UA was confirmed using the UHPLC-DAD-MS method; 196 mg (41%).

#### Synthesis of mixture of 8-hydroxy-6-oxo-6H-benzo[c]chromen-3-yl 2-(4-isobutylphenyl)propanoate and 3-hydroxy-6-oxo-6H-benzo[c]chromen-8-yl 2-(4-isobutylphenyl)propanoate (Mix 3a/3b)

To a magnetically stirred at 50 °C suspension of UA (0.050 g; 0.22 mmol, 1.0 eqv) and triethylamine (0.036 mL; 0.26 mmol, 1.2 eqv) in dioxane (10 mL) solution of ibuprofen chloride (0.049 g; 0.22 mmol, 1.0 eqv) in CH_2_Cl_2_ (1 mL) was added. After 2 h the reaction mixture was concentrated under reduced pressure. To residue ethyl acetate (30 mL) and water (15 mL) were added and acidified to pH 2–3 with 3% HCl_aq_ solution (2 mL). After separation of the phases the water layer was additionally extracted with ethyl acetate (2 × 20 mL). The combined organic phases were washed with brine (15 mL) and dried over anhydrous Na_2_SO_4_. After evaporation of the solvent under reduced pressure the product was isolated using column chromatography on silica gel and CH_2_Cl_2_:MeOH mixture (0–8% MeOH) as an eluent. After purification the product was obtained as an solidifying oil; 40 mg (44%). Mp = 98.3–99.2 °C. ^1^H NMR (500 MHz, CDCl_3_) δ 7.87 (d, *J* = 2.4 Hz, 1H), 7.78 (d, *J* = 8.8 Hz, 1H), 7.75 (d, *J* = 8.6 Hz, 1H), 7.68 (d, *J* = 8.8 Hz, 1H), 7.58 (d, *J* = 8.8 Hz, 1H), 7.43 (d, *J* = 2.5 Hz, 1H), 7.40–7.30 (m, 5H), 7.19 (d, *J* = 7.7 Hz, 4H), 7.12 (dd, *J* = 8.8, 2.6 Hz, 1H), 6.94 (d, *J* = 2.0 Hz, 1H), 6.91 (dd, *J* = 8.6, 2.2 Hz, 1H), 6.63 (dd, *J* = 8.7, 2.3 Hz, 1H), 6.52 (d, *J* = 2.3 Hz, 1H), 4.06–3.98 (m, 2H), 2.50 (d, *J* = 7.2 Hz, 4H), 1.94–1.81 (m, 2H), 1.66 (dd, *J* = 7.1, 4.3 Hz, 6H), 0.93 (d, *J* = 6.6 Hz, 12H). ^13^C NMR (125 MHz, CDCl_3_) δ 174.74, 174.72, 161.03, 160.93, 158.40, 156.87, 151.79, 150.85, 150.53, 149.74, 141.41, 141.38, 136.71, 136.59, 133.19, 129.90, 129.87, 129.56, 128.93, 127.34, 127.32, 126.79, 124.06, 123.59, 123.29, 123.07, 122.78, 122.74, 121.55, 120.71, 118.31, 116.26, 114.97, 113.48, 110.95, 110.14, 103.87, 45.45, 45.42, 45.19, 30.33, 30.31, 22.57, 22.55, 18.58, 18.54. MS (m/z) [M]^+^: 417.

#### Synthesis of mixture of 8-hydroxy-6-oxo-6H-benzo[c]chromen-3-yl 2-((2,3-dimethylphenyl)amino)benzoate and 3-hydroxy-6-oxo-6H-benzo[c]chromen-8-yl 2-((2,3-dimethylphenyl)amino)benzoate (Mix 4a/4b)

To a magnetically stirred at room temperature suspension of UA (0.200 g; 0.88 mmol, 1.0 eqv) in a mixture of dioxane (200 mL) and dichloromethane (100 mL) mefenamic acid (0.212 g; 0.88 mmol, 1.0 eqv), DCC (0.217 g; 1.05 mmol; 1.2 eqv) and DMAP (21.40 mg; 0.18 mmol; 0.2 eqv) were subsequently added. After 24 h the reaction mixture was concentrated under reduced pressure. The residual oil was purified using column chromatography on silica gel and CH_2_Cl_2_:MeOH mixture (0–8% MeOH) as an eluent. After purification the product was obtained as pale yellow solid; 105 mg (26%). Mp = 188.0–189.5 °C. ^1^H NMR (500 MHz, CDCl_3_) δ 9.09 (s, 1H), 9.07 (s, 1H), 8.21 (d, *J* = 6.9 Hz, 2H), 8.15 (d, *J* = 2.3 Hz, 1H), 7.97 (d, *J* = 9.3 Hz, 1H), 7.93 (d, *J* = 8.7 Hz, 1H), 7.83 (d, *J* = 9.1 Hz, 1H), 7.74 (d, *J* = 9.3 Hz, 1H), 7.64 (dd, *J* = 7.6, 2.1 Hz, 1H), 7.60 (d, *J* = 2.4 Hz, 1H), 7.35 (t, *J* = 7.9 Hz, 2H), 7.27–7.24 (m, 1H), 7.23–7.16 (m, 4H) 7.13 (t, *J* = 7.8 Hz, 2H), 7.06 (d, *J* = 7.4 Hz, 2H), 6.81–6.74 (m, 5H), 6.71 (d, *J* = 2.1 Hz, 1H), 2.33 (s, 6H), 2.18 (s, 6H). ^13^C NMR (125 MHz, CDCl_3_) δ 167.97 (s, *J* = 5.7 Hz), 167.92, 161.16, 158.52, 156.98, 152.07, 151.05–150.95 (m, *J* = 6.2 Hz), 150.91, 150.86, 150.79, 149.91, 138.54, 138.14, 135.82, 133.24, 133.08 (s, *J* = 10.4 Hz), 132.99, 132.18, 129.56, 127.58 (s, *J* = 4.5 Hz), 127.55, 127.07, 126.23, 124.18, 123.83 (s, *J* = 12.3 Hz), 123.74, 123.50, 123.34, 123.19, 122.89, 121.79, 120.99, 118.97, 116.52 (s, *J* = 19.2 Hz), 116.37, 115.14, 114.07, 113.56, 111.60, 110.45, 108.82 (s, *J* = 13.5 Hz), 108.72, 104.10, 66.06, 33.98, 25.65, 24.98, 20.74, 15.36, 14.21. MS (m/z) [M]^+^: 452.

#### Synthesis of mixture of 8-hydroxy-6-oxo-6H-benzo[c]chromen-3-yl 2-(2-((2,6-dichlorophenyl)amino)phenyl)acetate and 3-hydroxy-6-oxo-6H-benzo[c]chromen-8-yl 2-(2-((2,6-dichlorophenyl)amino)phenyl)acetate (Mix 5a/5b)

To a magnetically stirred at room temperature suspension of UA (0.100 g; 0.44 mmol, 1.0 eqv) in a mixture of dioxane (100 mL) and dichloromethane (50 mL) diclofenac (0.260 g; 0.88 mmol, 2.0 eqv), DCC (0.182 g; 0.88 mmol; 2.0 eqv) and DMAP (21.40 mg; 0.18 mmol; 0.4 eqv) were subsequently added. After 24 h the reaction mixture was concentrated under reduced pressure. The residual oil was purified using column chromatography on silica gel and CH_2_Cl_2_:MeOH mixture (0–8% MeOH) as an eluent. After purification the product was obtained as pale beige solid; 33 mg (14%). Mp = 188.8–191.0 °C. ^1^H NMR (500 MHz, CDCl_3_) δ 7.97 (d, *J* = 2.6 Hz, 1H), 7.93 (d, *J* = 8.9 Hz, 1H), 7.90 (d, *J* = 8.8 Hz, 1H), 7.89 (d, *J* = 8.9 Hz, 1H), 7.78 (d, *J* = 8.8 Hz, 1H), 7.62 (d, *J* = 2.7 Hz, 1H), 7.46 (dd, *J* = 8.8, 2.6 Hz, 1H), 7.31–7.26 (m, 7H), 7.13 (dd, *J* = 11.1, 4.4 Hz, 2H), 7.09 (d, *J* = 2.3 Hz, 1H), 7.03 (dd, *J* = 8.7, 2.3 Hz, 1H), 6.99–6.92 (m, 4H), 6.79 (dd, *J* = 8.7, 2.4 Hz, 1H), 6.74 (d, *J* = 2.4 Hz, 1H), 6.53 (d, *J* = 8.0 Hz, 2H), 4.03 (d, *J* = 5.5 Hz, 4H). ^13^C NMR (125 MHz, CDCl_3_) δ 170.51, 170.41, 161.57, 161.32, 159.57, 158.01, 152.16, 150.46, 150.21, 149.31, 142.59, 142.57, 142.48, 142.47, 137.52, 137.46, 133.60, 130.94, 129.42, 129.40, 129.36, 129.34, 128.92, 128.77, 128.34, 126.38, 126.30, 124.60, 124.28, 124.19, 123.86, 123.59, 123.43, 123.40, 123.37, 123.33, 122.76, 122.52, 122.47, 122.32, 122.28, 122.26, 121.60, 120.42, 118.45, 118.21, 116.53, 114.36, 113.43, 110.75, 109.54, 103.49, 49.54, 49.39, 49.37, 49.20, 49.03, 48.86, 48.69, 48.52, 38.35, 38.26. MS (m/z) [M]^+^: 507.

#### Synthesis of mixture of 8-hydroxy-6-oxo-6H-benzo[c]chromen-3-yl 2-acetoxybenzoate and 3-hydroxy-6-oxo-6H-benzo[c]chromen-8-yl 2-acetoxybenzoate (Mix 6a/6b)

To a magnetically stirred at 50 °C suspension of UA (0.100 g; 0.44 mmol, 1.0 eqv) and triethylamine (0.072 mL; 0.53 mmol, 1.2 eqv) in dioxane (50 mL) solution of aspirin chloride (0.087 g; 0.44 mmol, 1.0 eqv) in CH_2_Cl_2_ (3 mL) was added. After 3 h the reaction mixture was concentrated under reduced pressure. The residual oil was purified using column chromatography on silica gel and CH_2_Cl_2_:MeOH mixture (0–2% MeOH) as an eluent. After purification the product was obtained as pale beige solid; 60 mg (35%). Mp = 176.8–178.0 °C. ^1^H NMR (500 MHz, CDCl_3_) δ 8.22–8.17 (m, 2H), 8.07 (d, *J* = 2.5 Hz, 1H), 7.98 (d, *J* = 8.8 Hz, 1H), 7.96 (d, *J* = 8.7 Hz, 1H), 7.91 (d, *J* = 8.8 Hz, 1H), 7.80 (d, *J* = 8.8 Hz, 1H), 7.66–7.61 (m, 3H), 7.56 (dd, *J* = 8.7, 2.5 Hz, 1H), 7.40–7.35 (m, 2H), 7.30 (dd, *J* = 8.7, 2.7 Hz, 1H), 7.18–7.14 (m, 3H), 7.13 (dd, *J* = 8.7, 2.3 Hz, 1H), 6.80 (dd, *J* = 8.7, 2.4 Hz, 1H), 6.76 (d, *J* = 2.4 Hz, 1H), 2.28 (s, 6H). ^13^C NMR (125 MHz, CDCl_3_) δ 170.08, 162.79, 162.75, 161.67, 161.41, 159.67, 158.13, 152.31, 151.32, 151.26, 150.56, 150.47, 149.44, 135.10, 135.05, 133.76, 132.23, 129.22, 126.46, 126.41, 126.39, 124.44, 124.16, 124.12, 124.01, 123.74, 123.04, 122.78, 122.74, 122.04, 121.93, 121.75, 120.69, 118.51, 116.73, 114.54, 113.59, 111.08, 109.72, 103.68, 73.38, 68.25, 61.34, 49.69, 49.52, 49.34, 49.17, 49.00, 20.99. MS (m/z) [M]^+^: 391.

#### Synthesis of 8-methoxy-6-oxo-6H-benzo[c]chromen-3-yl 2-(4-isobutylphenyl)propanoate (7)

To a magnetically stirred at 50 °C solution of **2** (0.030 g; 0.12 mmol, 1.0 eqv) in a mixture of dioxane (10 mL) and dichloromethane (5 mL) triethylamine (0.052 mL; 0.36 mmol, 3.0 eqv) and next solution of ibuprofen chloride (0.058 g; 0.24 mmol, 2.0 eqv) in CH_2_Cl_2_ (1 mL) were added. After 1 h the reaction mixture was concentrated under reduced pressure. To residue dichloromethane (20 mL) and water (8 mL) were added and acidified to pH 2–3 with 3% HCl_aq_ solution (1 mL). After separation of the phases the water layer was additionally extracted dichloromethane (10 mL). The combined organic phases were washed with water (8 mL) and dried over anhydrous MgSO_4_. After evaporation of the solvent under reduced pressure the product was isolated using column chromatography on silica gel and CH_2_Cl_2_ as an eluent. After purification the product was obtained as white solid; 52 mg (98%). Mp = 114.4–115.7 °C. ^1^H NMR (500 MHz, CDCl_3_) δ 7.94 (d, *J* = 8.9 Hz, 1H), 7.90 (d, *J* = 8.7 Hz, 1H), 7.76 (d, *J* = 2.8 Hz, 1H), 7.36 (dd, *J* = 8.8, 2.8 Hz, 1H), 7.29 (d, *J* = 8.0 Hz, 2H), 7.15 (d, *J* = 8.2 Hz, 2H), 7.02 (d, *J* = 2.2 Hz, 1H), 6.97 (dd, *J* = 8.7, 2.3 Hz, 1H), 3.95 (q, *J* = 7.1 Hz, 1H), 3.91 (s, 3H), 2.47 (d, *J* = 7.2 Hz, 2H), 1.93–1.83 (m, 1H), 1.61 (d, *J* = 7.2 Hz, 3H), 0.90 (d, *J* = 6.6 Hz, 6H). ^13^C NMR (125 MHz, CDCl_3_) δ 172.99, 161.12, 160.12, 151.45, 150.86, 141.19, 137.03, 129.77, 127.83, 127.32, 124.56, 123.53, 122.95, 122.08, 118.36, 116.10, 111.38, 110.95, 55.94, 45.41, 45.19, 30.33, 22.55, 18.61. MS (m/z) [M]^+^: 431.

#### Synthesis of 8-methoxy-6-oxo-6H-benzo[c]chromen-3-yl 2-((2,3-dimethylphenyl)amino)benzoate (8)

To a magnetically stirred at room temperature suspension of **2** (0.050 g; 0.21 mmol, 1.0 eqv) in a mixture of dioxane (20 mL) and dichloromethane (10 mL) mefenamic acid (0.050 g; 0.21 mmol, 1.0 eqv), DCC (0.052 g; 0.25 mmol; 1.2 eqv) and DMAP (5.13 mg; 0.042 mmol; 0.2 eqv) were subsequently added. After 24 h the reaction mixture was concentrated under reduced pressure. The residual oil was purified using column chromatography on silica gel and CH_2_Cl_2_ as an eluent. After purification the product was obtained as white solid; 50 mg (51%). Mp = 186.7–189.2 °C. ^1^H NMR (500 MHz, CDCl_3_) δ 9.14 (s, 1H), 8.20 (dd, *J* = 8.1, 1.4 Hz, 1H), 8.04 (d, *J* = 8.7 Hz, 1H), 8.01 (s, 1H), 7.82 (d, *J* = 2.8 Hz, 1H), 7.42 (dd, *J* = 8.8, 2.8 Hz, 1H), 7.34 (ddd, *J* = 8.6, 7.1, 1.4 Hz, 1H), 7.29 (d, *J* = 2.3 Hz, 1H), 7.24 (dd, *J* = 8.6, 2.3 Hz, 1H), 7.17 (d, *J* = 7.6 Hz, 1H), 7.12 (t, *J* = 7.7 Hz, 1H), 7.05 (d, *J* = 7.3 Hz, 1H), 6.81 (dd, *J* = 8.6, 0.7 Hz, 1H), 6.76 (ddd, *J* = 8.1, 7.1, 1.1 Hz, 1H), 3.95 (s, 3H), 2.33 (s, 3H), 2.17 (s, 3H). ^13^C NMR (125 MHz, CDCl_3_) δ 166.93, 161.06, 160.03, 151.29, 150.90, 150.53, 138.35, 138.18, 135.33, 132.69, 131.91, 127.76, 127.22, 126.02, 124.48, 123.42 (d, *J* = 7.7 Hz), 123.00, 121.99, 118.84, 116.27, 116.10, 113.86, 111.46, 111.30, 109.01, 55.83, 34.93, 25.46, 24.70, 20.62, 14.02; . MS (m/z) [M]^+^: 466.

#### Synthesis of 8-methoxy-6-oxo-6H-benzo[c]chromen-3-yl 2-(2-((2,6-dichlorophenyl)amino)phenyl)acetate (9)

To a magnetically stirred at room temperature suspension of **2** (0.050 g; 0.21 mmol, 1.0 eqv) in a mixture of dioxane (20 mL) and dichloromethane (10 mL) diclofenac (0.124 g; 0.42 mmol, 2.0 eqv), DCC (0.087 g; 0.42 mmol; 2.0 eqv) and DMAP (10.30 mg; 0.084 mmol; 0.4 eqv) were subsequently added. After 24 h the reaction mixture was concentrated under reduced pressure. The residual oil was purified using column chromatography on silica gel and CH_2_Cl_2_ as an eluent. After purification the product was obtained as pale beige solid; 14 mg (13%). Mp = 207.3–208.5 °C. ^1^H NMR (300 MHz, CDCl_3_) δ 7.99 (d, *J* = 4.9 Hz, 1H), 7.96 (d, *J* = 4.7 Hz, 1H), 7.80 (d, *J* = 2.8 Hz, 1H), 7.40 (dd, *J* = 8.9, 2.8 Hz, 1H), 7.35–7.32 (m, 3H), 7.22–7.16 (m, 1H), 7.17 (d, *J* = 2.1 Hz, 1H), 7.11 (dd, *J* = 8.7, 2.4 Hz, 1H), 6.96–7.07 (m, 2H), 6.61 (d, *J* = 8.0 Hz, 1H), 4.10 (s, 2H), 3.94 (s, 3H). ^13^C NMR (75 MHz, CDCl_3_) δ 170.30, 160.97, 160.09, 150.94, 150.78, 142.73, 137.70, 131.08, 129.49, 128.90, 128.47, 127.63, 124.48, 124.24, 123.57, 123.46, 123.01, 122.43, 122.04, 118.68, 118.30, 116.32, 111.30, 111.00, 55.84, 38.57. MS (m/z) [M]^+^: 521.

#### Synthesis of 8-methoxy-6-oxo-6H-benzo[c]chromen-3-yl 2-acetoxybenzoate (10)

To a magnetically stirred at 50 °C solution of **2** (0.060 g; 0.25 mmol, 1.0 eqv) in a mixture of dioxane (20 mL) and dichloromethane (10 mL) triethylamine (0.103 mL; 0.75 mmol, 3.0 eqv) and next solution of aspirin chloride (0.099 g; 0.50 mmol, 2.0 eqv) in CH_2_Cl_2_ (2 mL) were added. After 3 h the reaction mixture was concentrated under reduced pressure. To residue dichloromethane (20 mL) and water (8 mL) were added and acidified to pH 2–3 with 3% HCl_aq_ solution (1 mL). After separation of the phases the water layer was additionally extracted dichloromethane (10 mL). The combined organic phases were washed with water (8 mL) and dried over anhydrous MgSO_4_. After evaporation of the solvent under reduced pressure the product was isolated using column chromatography on silica gel and CH_2_Cl_2_ as an eluent. After purification the product was obtained as white solid; 60 mg (60%). Mp = 192.6–193.5 °C. ^1^H NMR (500 MHz, CDCl_3_) δ 8.20 (dd, *J* = 7.9, 1.7 Hz, 1H), 8.01–7.96 (m, 2H), 7.76 (d, *J* = 2.8 Hz, 1H), 7.64 (ddd, *J* = 8.1, 7.6, 1.7 Hz, 1H), 7.40–7.36 (m, 2H), 7.20 (d, *J* = 2.3 Hz, 1H), 7.16 (ddd, *J* = 8.7, 4.0, 1.7 Hz, 2H), 3.91 (s, 3H), 2.29 (s, 3H). ^13^C NMR (125 MHz, CDCl_3_) δ 169.98, 162.66, 161.22, 160.16, 151.31, 150.93, 150.82, 135.06, 132.25, 127.69, 126.38, 124.56, 124.15, 123.58, 123.24, 122.05,121.96, 118.56, 116.43, 111.37, 111.18, 55.86, 49.62, 49.45, 49.28, 21.02. MS (m/z) [M]^+^: 405.

NMR spectra of UADs with methoxy group in 8 position and esterified group in 3- position (Fig. S2) are presented in Figs. S3–S6.

### UHPLC-DAD-MS

Unless otherwise specified, methanol was used to dissolve samples before UHPLC-DAD-MS analysis. The experiments were conducted on a UHPLC-3000 RS system (Dionex, Leipzig, Germany), outfitted with diode array detector coupled with AmaZon SL ion trap mass spectrometer with an ESI interface (Bruker Daltonik GmbH, Bremen, Germany). The injection volume was 10 µL. The compounds were separated using Kinetex XB-C_18_ (Phenomenex, Torrance, CA, 150 mm × 2.1 mm, particle size 1.7 µm) analytical column at temperature 25 °C. The elution was performed in gradient system using mobile phase A (H2O:formic acid (99.9:0.1, *v/v*)), and mobile phase B (acetonitrile:formic acid (99.9:0.1, *v/v*)), starting from with the 35% of mobile phase B, 35–95% for 20 min and additional 5 min with constant, 95% phase B concentration. The flow rate was set to 0.3 ml/min. UV detection of UADs was accomplished at λ = 305 nm. The parameters of ESI source were as follows: nebuliser pressure: 40 psi, drying gas flow rate: 9 L/min, dry gas temperature 300 °C, and capillary voltage: 4.5 kV. The mass spectra were registered by scanning from m/z 70 to 2200. Identification of compounds was based on the determination of their molecular mass, UV–Vis spectra and fragmentation profile.

### NMR

The NMR spectra were recorded on a Bruker AVANCE (Bruker, Karlsruhe, Germany) spectrometer operating at 300 or 500 MHz for ^1^H NMR and at 125 MHz or 75 MHz for ^13^C NMR. The spectra were measured in CDCl_3_ and are given as δ values (in ppm) relative to TMS, and coupling constants (J) are reported in Hz.

### Stability assays

The stability assays under several stress conditions were performed according to modified, previously described protocol^29^. Detailed information regarding stability assays’ conditions are described in Supporting Information. Samples were dissolved in DMSO to obtain 20 mg/ml concentration and added to aliquots to achieve 80 mg/ml concentration at the start of incubation. All experiments were performed in triplicates (n = 3). The degree of degradation was calculated as follows:

$$Degradation \left( \% \right) = \left[ {1 - \left( {\frac{Peak\;area\;of\;UAD\;in\;aliqout}{{Peak\;area\;of\;UAD\;in\;control}}} \right)} \right] \times 100.$$

### THP-1 derived macrophages

THP-1 human monocytic cells (DSMZ, Braunschweig, Germany) were studied between passages 4 and 19. Monocytes were differentiated into THP-1 derived macrophages according to modified, previously described protocol^17^. THP-1 monocytes were seeded in 24-well plates at a density of 400,000 cells per well. Cells were incubated at 37 °C under humidified 5% CO_2_ in the culture medium containing RPMI 1640 without phenol red supplemented with 10% FBS and 2 mM glutamine. In order to differentiate THP-1 monocytes into macrophages, cells were treated with 25 ng/mL PMA followed by 48 h incubation, medium change and an additional 24 h resting. Selected UADs were added to THP-1 derived macrophages at concentrations 2–50 µM and left for 1-h resting. Next, cells were treated with 10 ng/ml LPS. Successful differentiation was confirmed by the presence of distinguishing macrophage markers such as: cell adherence, changes in morphology and response to bacterial LPS stimulation and, consequently, TNF-α secretion.

### MTT assay

THP-1 monocytes were seeded in 24-well plates at a density of 400,000 cells per well and differentiated into THP-1 derived macrophages according to the method described above. After treatment with the test compounds for 1 h and subsequent stimulation with LPS for 3 or 24 h, cells were washed with warm PBS. Afterwards, a 1 mL of solution of 0.5 mg/mL MTT in culture medium was added, and macrophages were incubated at 37 °C under humidified 5% CO_2_ for 30 min. After medium removal, crystals were dissolved in 400 µL DMSO, 200 µL of solution was transferred to 96-well plate and the absorbance of the solution was measured at λ = 570 nm with the correction to 630 nm using a microplate reader. The relative cell viability was shown as the experimental group's ratio to stimulated cells.

### Neutral red uptake (NRU) assay

NRU assay was performed according to modified, previously described protocol^39^. 40 mg of neutral red dye in was dissolved in 10 mL distilled water to obtain 4 mg/ mL neutral red stock solution. Subsequently, 140 µL of neutral red stock solution was added to 14 ml of culture medium and sterile filtered in order to obtain 40 µg/mL neutral red medium. THP-1 monocytes were seeded in 24-well plates at a density of 400,000 cells per well and differentiated into THP-1 derived macrophages according to the method described above. After treatment with the test compounds for 1 h and subsequent stimulation with LPS for 3 or 24 h, cells were washed with warm PBS. Afterwards, a 500 µL of warm 40 µg/mL neutral red medium was added and macrophages were incubated at 37 °C under humidified 5% CO_2_ for 2 h. After this time cells were washed with PBS and 500 µL of neutral red destain solution was added and the plate was placed in plate shaker for at least 15 min. The absorbance of the solution was measured at λ = 530 nm with the correction to 645 nm using a microplate reader. The relative cell viability was shown as the experimental group's ratio to stimulated cells. The experiment was performed in triplicate (n = 3).

### Enzyme‑linked immunosorbent assay (ELISA)

After treatment with the test compounds for 1 h and subsequent stimulation with LPS for 3 h (for TNF-α secretion measurements) or 24 h (for IL-10 and IL-6 secretion measurements) supernatants were collected. The secretion of cytokines levels was measured by ELISA kits according to the manufacturer’s instruction (Human TNF-α, Human IL-10 and Human IL-6 ELISA kits, BD Biosciences, Franklin Lakes, NJ, USA). The relative cytokines levels secretion was shown as the experimental group’s ratio to LPS stimulated cells.

### Propidium iodide (PI) staining

THP-1 monocytes were seeded in 24-well plates at a density of 400,000. Selected UADs (2–50 µM), control (0.25% DMSO in PBS), UA (50 µM) and parthenolide (25 µM) as a control were added to THP-1 monocytes and left for 24-h incubation at 37 °C under humidified 5% CO_2_ in the culture medium containing RPMI 1640 without phenol red supplemented with 10% FBS and 2 mM glutamine. After that time, cell suspensions were transferred to Eppendorf tubes, centrifuged, stained with propidium iodide (PI) and analyzed using FACS (MACSQuant® Analyze, Miltenyi Biotec, Bergisch Gladbach, Germany). The cell viability was reported as the percentage of PI(−) cells.

### Statistical analysis

All in vitro experiments were performed in triplicates (n = 3), at least from 3 independent experiments, if not otherwise stated. The significance of obtained differences was determined using one-way ANOVA, and post hoc Dunnett’s test was used to compare results with control groups. Tukey’s post hoc test was performed to compare differences in inhibitory activity between groups. The *p* value < 0.05 was considered statistically significant. Statistica 13 software was used to perform all analyses.

## Supplementary Information

Supplementary Information.

## Data Availability

All data generated or analysed during this study are included in this published article (and its supplementary information files).

## Abbreviations

AhR: Aryl hydrocarbon receptor
API: Active pharmaceutical ingredient
EA: Ellagic acid
ETs: Ellagitannins
IL-6: Interleukin-6
IL-10: Interleukin-10
LPS: Lipopolysaccharide
NSAIDs: Non-steroidal anti-inflammatory drugs
PBMC: Peripheral blood mononuclear cells
TNF-α: Tumor necrosis factor α
UA: Urolithin A
UADs: Urolithin A derivatives
UGT: Uridine 5′-diphospho-glucuronosyltransferase
