# Author Correction: Conjugates of urolithin A with NSAIDs, their stability, cytotoxicity, and anti-inflammatory potential

**DOI:** 10.1038/s41598-022-22926-2

**Published:** 2022-11-02

**Authors:** Maciej Korczak, Piotr Roszkowski, Sebastian Granica, Jakub P. Piwowarski

**Affiliations:** 1 Microbiota Lab, Department of Pharmacognosy and Molecular Basis of Phytotherapy, Medical University of Warsaw, ul. Banacha 1, 02‑097 Warsaw, Poland; 2 Laboratory of Natural Products Chemistry, Faculty of Chemistry, University of Warsaw, Warsaw, Poland

Correction to: *Scientific Reports* 10.1038/s41598-022-15870-8, published online 08 July 2022

The original version of this Article contained an error in the grant number stated in the Acknowledgments section.

“Project financially supported by Polish National Science Centre research Grant Preludium Bis No. UMO-2019/35/B/NZ8/01388.”

now reads:

“Project financially supported by Polish National Science Centre research Grant Preludium Bis No. UMO-2019/35/O/NZ7/00619.”

The original Article has been corrected.

