# Supplementary material for: Conjugates of urolithin A with NSAIDs, their stability, cytotoxicity, and anti-inflammatory potential
Source: Sci Rep. 2022 Jul 8;12:11676. doi: 10.1038/s41598-022-15870-8 (PMC9270351; doi:10.1038/s41598-022-15870-8)
Supplement: Supplementary file 1 — Supplementary Information. [file 41598_2022_15870_MOESM1_ESM.pdf]

Supplementary information

Conjugates of urolithin A with NSAIDs, their stability, cytotoxicity, and anti-inflammatory potential.

Maciej Korczak[1], Piotr Roszkowski[2], Sebastian Granica[1], Jakub P. Piwowarski[1*]

[1]Microbiota Lab, Department of Pharmacognosy and Molecular Basis of Phytotherapy, Medical University of Warsaw, Warsaw, Poland

[2]Faculty of Chemistry, Warsaw University, Warsaw, Poland

Stability assay performance 2
Figure S1. 3
Figure S2 4
Figure S3. 5
Figure S4. 5
Figure S5. 6
Figure S6. 6
Figure S7. 7
Table S1. 8
Table S2. 8
Table S3. 9
Table S4. 10
Table S5. 11
Table S7. 12
Table S8. 13

Stability assay performance

Control experiments. UADs dissolved in DMSO were added to deionised water, mixed thoroughly and, immediately, samples were taken, added to 0.2% formic acid in acetonitrile and subjected to UHPLC-DAD-MS analysis. In experiments evaluating stability in human plasma, UADs dissolved in DMSO were added to plasma, mixed thoroughly and, immediately, samples were taken, added to 0.2% formic acid in acetonitrile, centrifuged at 13000 RPM for 10 minutes and supernatants were subjected to UHPLC-DAD-MS analysis.

Acid hydrolysis. UADs dissolved in DMSO were added to HCl (1M), mixed thoroughly and kept at 37 °C for 24h or 80 °C for 12h. Samples were taken, added to NaOH (1M), mixed with 0.2% formic acid in acetonitrile and subjected to UHPLC-DAD-MS analysis.

Base hydrolysis. UADs dissolved in DMSO were added to NaOH (1M), mixed thoroughly and kept at 37 °C for 24h or 80 °C for 12h. Samples were taken, added to HCl (1M), mixed with 0.2% formic acid in acetonitrile and subjected to UHPLC-DAD-MS analysis.

Thermal degradation. UADs dissolved in DMSO were added to deionised water, mixed thoroughly and kept at room temperature for 24h, 37 °C for 24h, 80 °C for 12h or -70 °C for 24h. Additionally, samples were exposed to a cycle consisting of incubation at -70 °C for 24h followed by 2h thawing in tap water at room temperature, subsequent freezing at -20 °C for 24h and 2h thawing in tap water at room temperature. Samples were taken, mixed with 0.2% formic acid in acetonitrile and subjected to UHPLC-DAD-MS analysis.

UV degradation. ADs dissolved in DMSO were added to deionised water, mixed thoroughly and kept at room temperature for 7h in plastic falcons and exposed to UV-C light (λ=254 nm, Osram G3058/OF lamp, Osram Licht AG, Munich, Germany). Samples were taken, mixed with 0.2% formic acid in acetonitrile and subjected to UHPLC-DAD-MS analysis.

Oxidative degradation. UADs dissolved in DMSO were added to 30% H_2O_2 and kept at 37 °C for 24h or 80 °C for 12h. Samples were taken, mixed with 0.2% formic acid in acetonitrile and subjected to UHPLC-DAD-MS analysis.

Stability in human plasma.

Human plasma was collected from three independent donors and obtained from the Warsaw Blood Donation Center. UADs dissolved in DMSO were added to plasma, mixed thoroughly and kept at 37 °C for 30, 90 and 240 minutes. Samples were taken, added to 0.2% formic acid in acetonitrile, centrifuged at 13000 RPM for 10 minutes and supernatants were subjected to UHPLC-DAD-MS analysis.

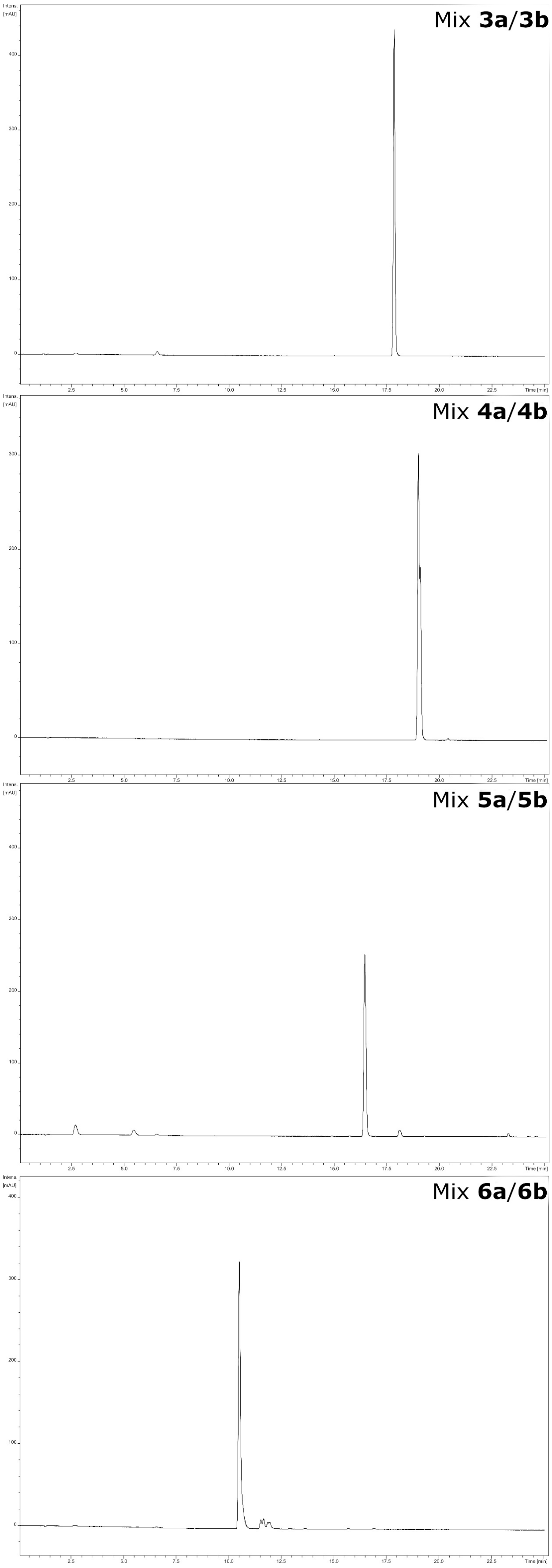

Figure S1. UHPLC profiles of **1** and synthesised UADs (λ= 305 nm)

7

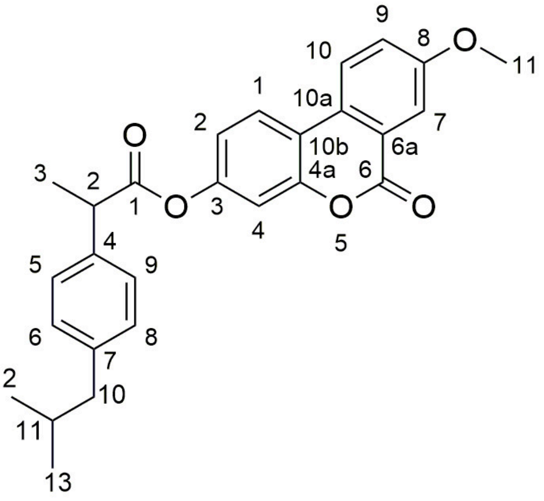

8

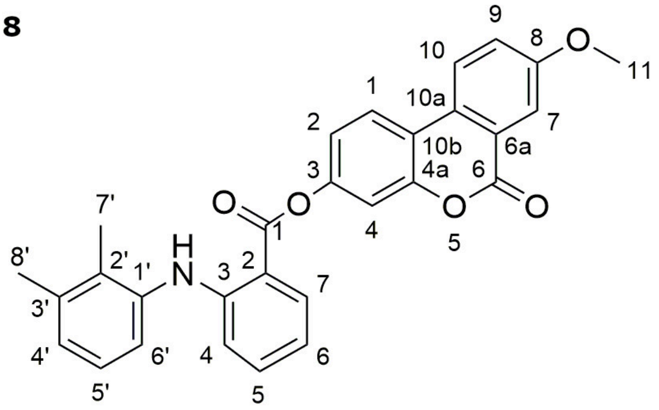

9

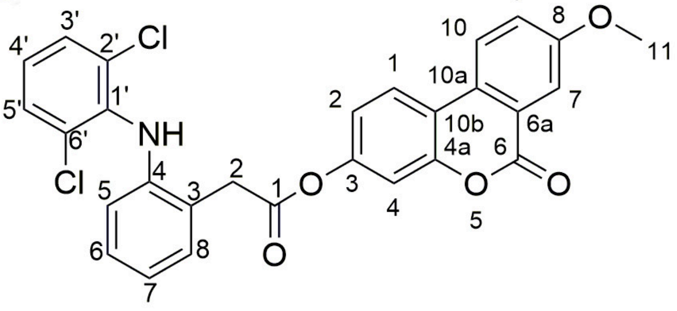

10

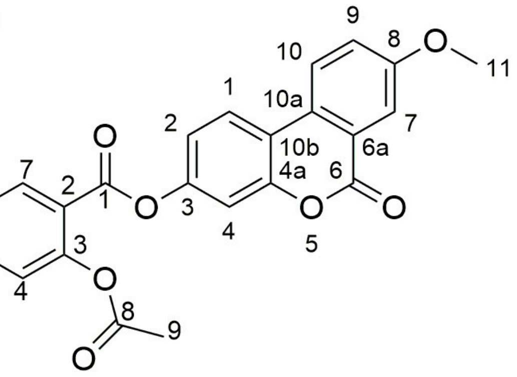

Figure S2 . Chemical structure of 3-NSAID-8-methoxy-UADs

7

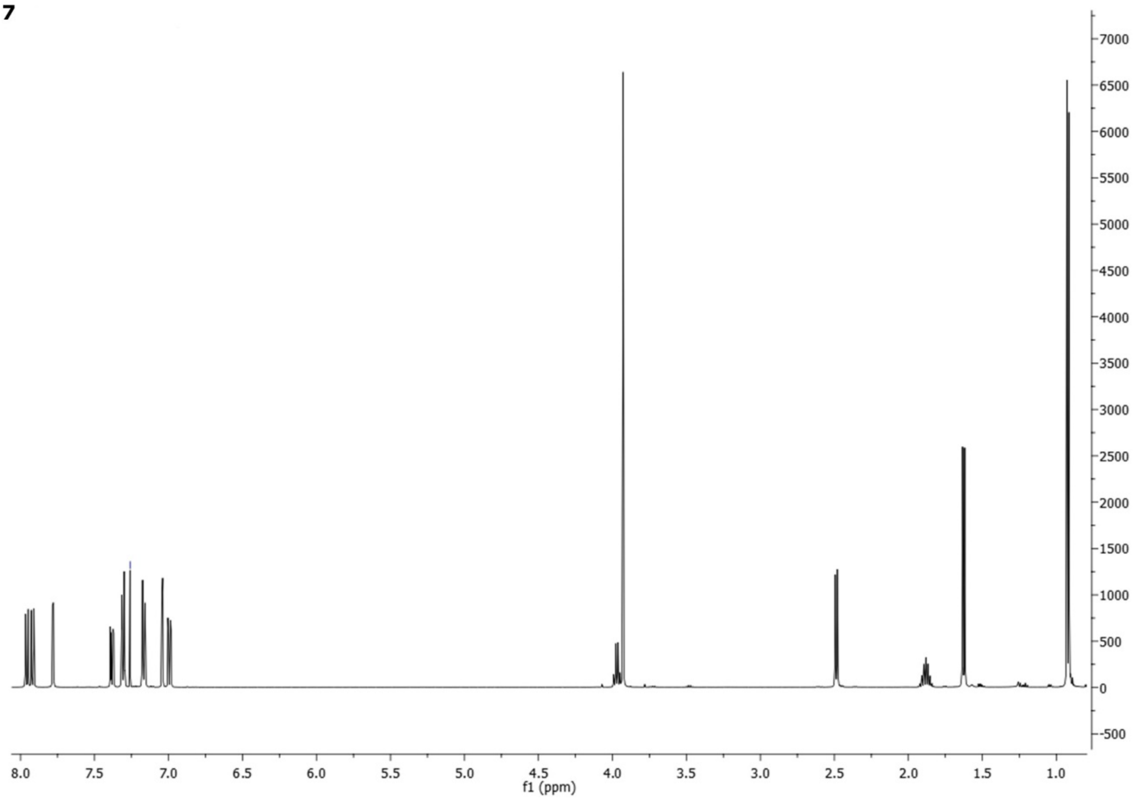

Figure S3. ^{1}H NMR spectrum of **7**

8

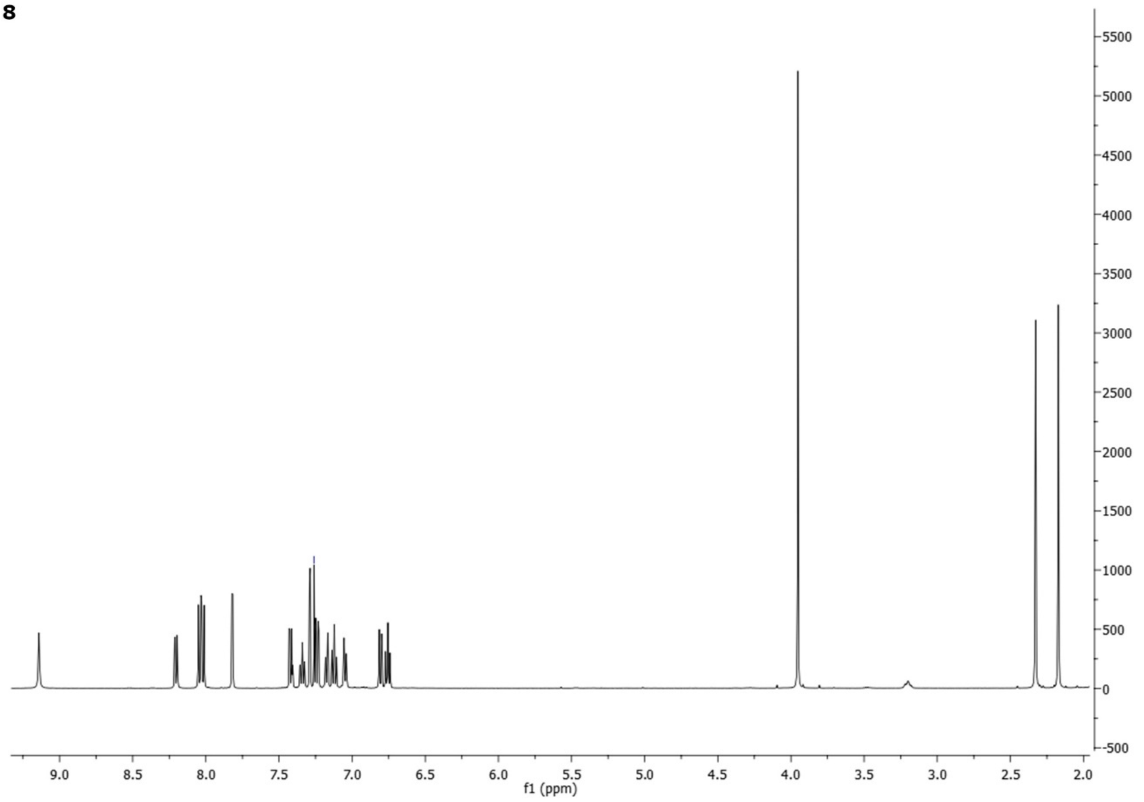

Figure S4. ^{1}H NMR spectrum of **8**

9

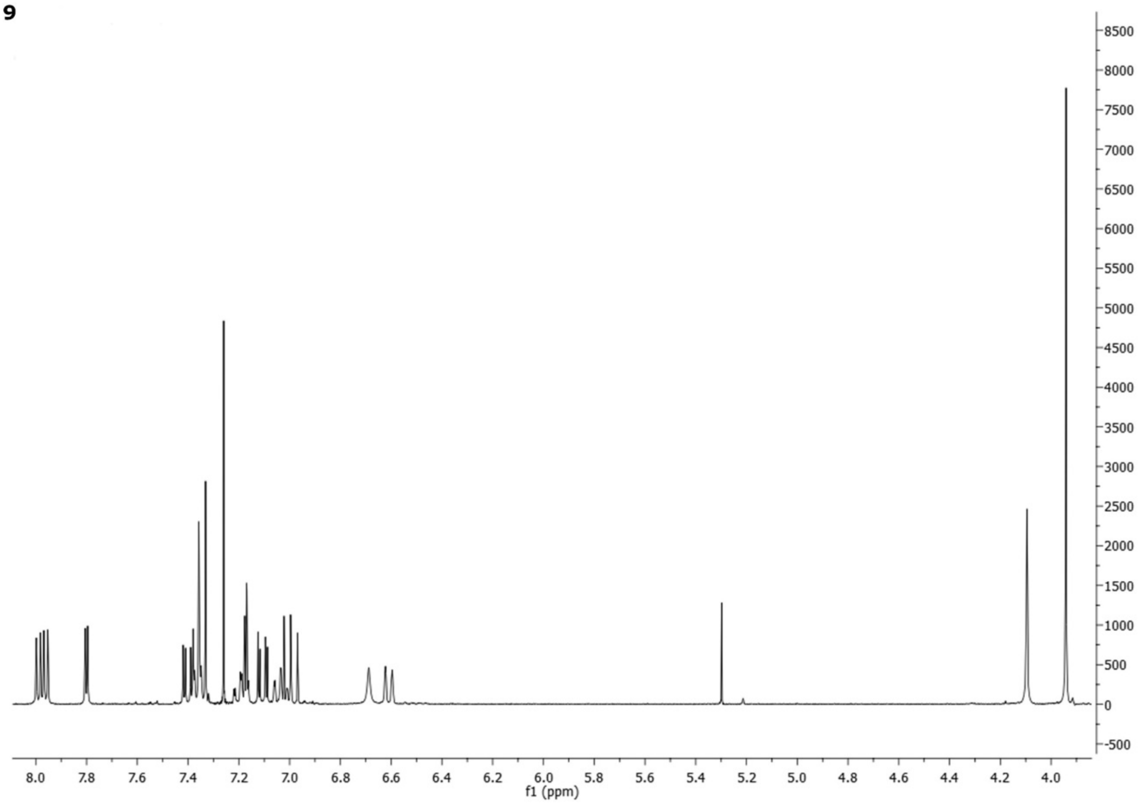

Figure S5. ^{1}H NMR spectrum of **9**

10

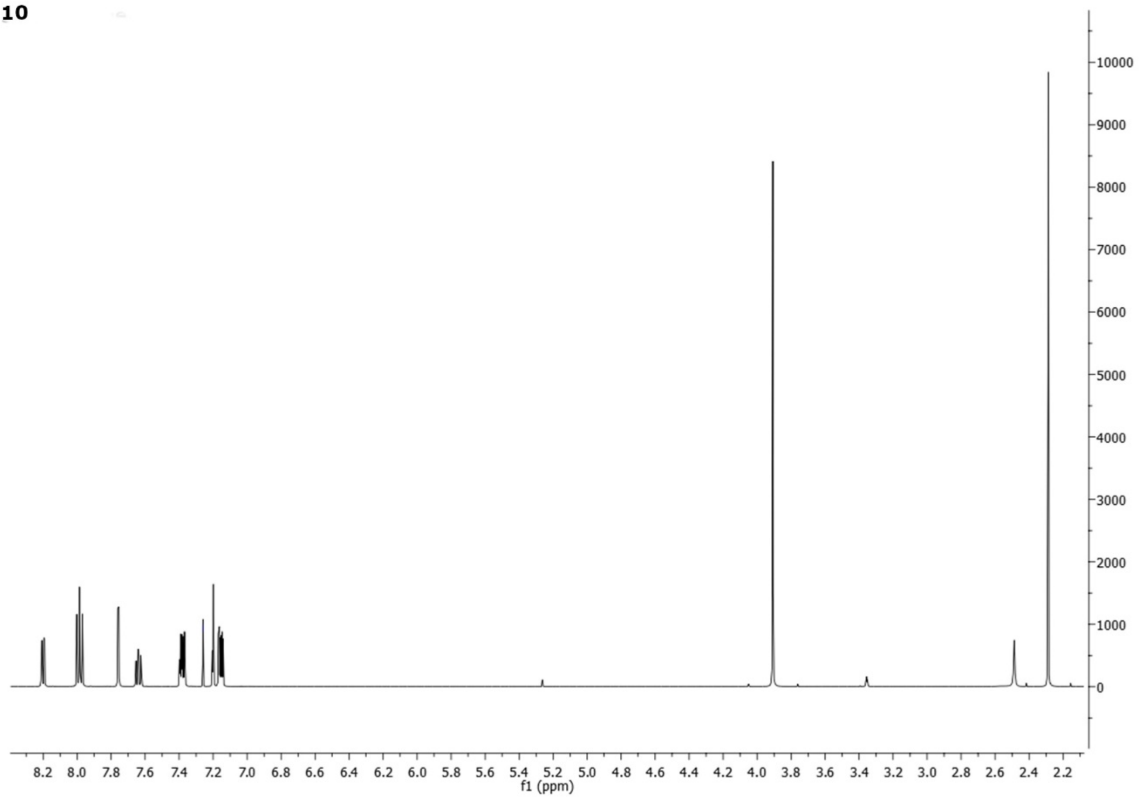

Figure S6. ^{1}H NMR spectrum of **10**

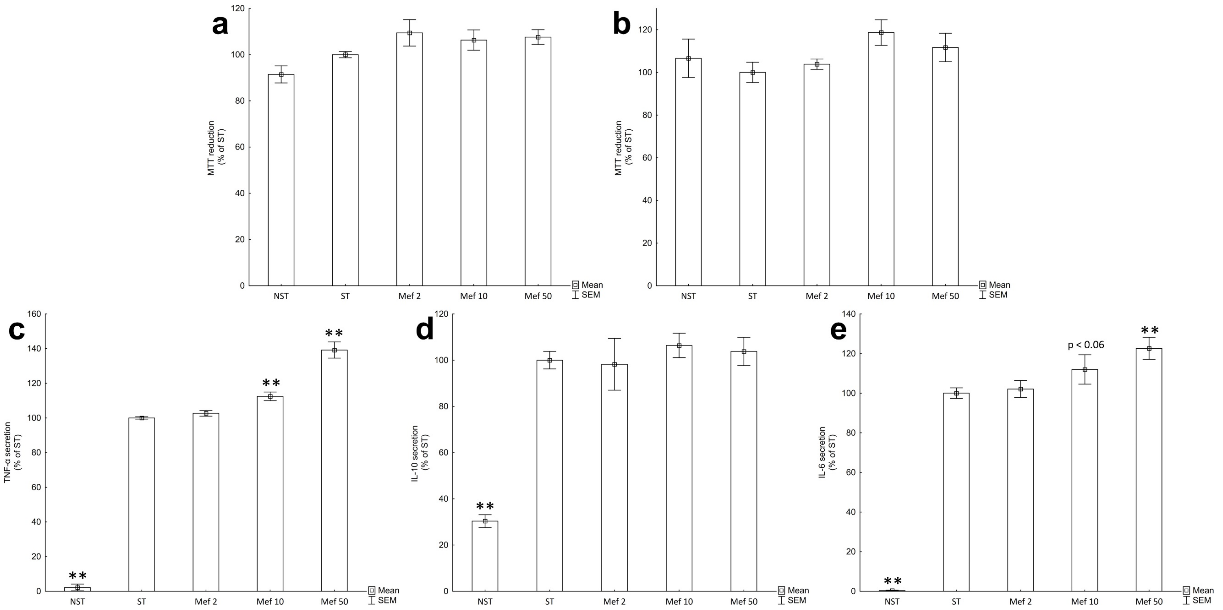

Figure S7. Evaluation of cytotoxic and anti-inflammatory activity of mefenamic acid using LPS stimulated THP-1 derived macrophages. (a) Effects of mefenamic acid on THP-1 derived macrophage viability measured using MTT assay after treatment with the test compounds for 1 h and subsequent stimulation with LPS for 3 h. (b) Effects of mefenamic acid on THP-1 derived macrophage viability measured using MTT assay after treatment with the test compounds for 1 h and subsequent stimulation with LPS for 24 h. (c) Effects of mefenamic acid on TNF-α secretion after treatment with the test compounds for 1 h and subsequent stimulation with LPS for 3 h. (d) Effects of mefenamic acid on IL-10 secretion after treatment with the test compounds for 1 h and subsequent stimulation with LPS for 24 h. (e) Effects of mefenamic acid on IL-6 secretion after treatment with the test compounds for 1 h and subsequent stimulation with LPS for 24 h. Data expressed as a mean±SEM. Numbers next graph labels indicate tested concentration (in μM). Statistical comparisons were made using the parametric method of one-way ANOVA, followed by the Dunnett's post hoc test. Statistical significance:**p< 0.01 versus ST group. NST – non-stimulated control, ST – LPS stimulated control, Mef – mefenamic acid.

	Viability (% of ST)	SEM	post hoc (Dunett) p (<ST)	p (>ST)
NST	91,46673	3,709197	0,166543	0,999692
ST	100	1,373162		
UA 2	100,3891	2,603849	0,907062	0,868297
UA 10	110,0242	5,546335	0,99991	0,091721
UA 50 µM	105,1696	2,525687	0,994954	0,450635
Mix **4a**/**4b** 2 µM	106,281	2,061571	0,997861	0,342196
Mix **4a**/**4b** 5 µM	106,1673	4,026725	0,997659	0,352808
Mix **4a**/**4b** 10 µM	102,4332	2,343859	0,968545	0,720091
Mix **4a**/**4b** 50 µM	39,26495	3,562343	0,000022	0,999963

post hoc (Tukey)	NST	ST	UA 50 µM	Mix **4a**/**4b** 2 µM	Mix **4a**/**4b** 5 µM	Mix **4a**/**4b** 10 µM		
NST								
ST	0,664729							
UA 2	0,60937	1						
UA 10	0,005047	0,45165	0,506566					
UA 50 µM	0,097931	0,971479	0,982403	0,980621				
Mix **4a**/**4b** 2 µM	0,053776	0,914156	0,939156	0,996551	1			
Mix **4a**/**4b** 5 µM	0,057315	0,922047	0,945413	0,995751	1	1		
Mix **4a**/**4b** 10 µM	0,328794	0,999856	0,999962	0,788094	0,999655	0,995819	0,996608	
Mix **4a**/**4b** 50 µM	0,000136	0,000136	0,000136	0,000136	0,000136	0,000136	0,000136	0,000136

Table S1. Post hoc analysis of effects of UA and Mix **4a**/**4b** on THP-1 derived macrophage viability measured using MTT assay 3 hours after stimulation. Cells viability presented as a ratio of the experimental group to LPS stimulated cells. ST group. NST – non-stimulated control, ST – LPS stimulated control

	Viability (% of ST)	SEM	post hoc (Dunett) p (<ST)	p (>ST)
NST	106,5945	8,995372	0,945726	0,721615
ST	100,0000	4,749834		
UA 50 µM	107,1844	2,987045	0,950537	0,706223
Mix **4a**/**4b** 2 µM	113,2348	7,804671	0,971350	0,614843
Mix **4a**/**4b** 5 µM	150,2666	18,84261	0,999973	0,002693
Mix **4a**/**4b** 10 µM	147,2280	7,333491	0,999973	0,004823
Mix **4a**/**4b** 50 µM	4,217185	0,190066	0,000089	0,999973

post hoc (Tukey)	NST	ST	UA 50 µM	Mix **4a**/**4b** 2 µM	Mix **4a**/**4b** 5 µM	Mix **4a**/**4b** 10 µM
NST						
ST	0,999744					
UA 50 µM	1,000000	0,999580				
Mix **4a**/**4b** 2 µM	0,999965	0,998053	0,999980			
Mix **4a**/**4b** 5 µM	0,179506	0,016282	0,191636	0,735161		
Mix **4a**/**4b** 10 µM	0,248497	0,028335	0,263756	0,803974	0,999991	
Mix **4a**/**4b** 50 µM	0,002947	0,006230	0,002757	0,001403	0,000136	0,000147

Table S2. Post hoc analysis of effects of UA and Mix **4a**/**4b** on THP-1 derived macrophage viability measured using MTT assay 24 hours after stimulation. Cells viability presented as a ratio of the experimental group to LPS stimulated cells. ST group. NST – non-stimulated control, ST – LPS stimulated control

	Viability (% of ST)	SEM	post hoc (Dunett) p (<ST)	p (>ST)
NST	110,9259	1,858783	0,999981	0,000065
ST	100,0000	0,975523		
UA 10 μM	102,4074	0,883277	0,996275	0,333029
UA 50 μM	102,7778	0,555556	0,998036	0,255639
Mix **4a/4b** 2 μM	102,1296	1,557649	0,994028	0,398113
Mix **4a/4b** 5 μM	102,9630	0,822981	0,998576	0,221591
Mix **4a/4b** 10 μM	104,5370	1,043466	0,999897	0,052107
Mix **4a/4b** 50 μM	91,20370	1,766554	0,000474	0,999981

post hoc (Tukey)	NST	ST	UA 10 μM	UA 50 μM	Mix **4a/4b** 2 μM	Mix **4a/4b** 5 μM	Mix **4a/4b** 10 μM
NST							
ST	0,000459						
UA 10 μM	0,004170	0,868225					
UA 50 μM	0,006179	0,769927	0,999999				
Mix **4a/4b** 2 μM	0,003123	0,923573	1,000000	0,999942			
Mix **4a/4b** 5 μM	0,007535	0,712945	0,999979	1,000000	0,999689		
Mix **4a/4b** 10 μM	0,040871	0,248682	0,923573	0,970601	0,868225	0,983979	
Mix **4a/4b** 50 μM	0,000175	0,003123	0,000386	0,000318	0,000459	0,000293	0,000192

Table S3. Post hoc analysis of effects of UA and Mix **4a**/**4b** on THP-1 derived macrophage viability measured using NRU assay 3 hours after stimulation. Cells viability presented as a ratio of the experimental group to LPS stimulated cells. ST group. NST – non-stimulated control, ST – LPS stimulated control

	Viability (% of ST)	SEM	post hoc (Dunett) p (<ST)	p (>ST)
NST	109,0261	1,992042	0,996146	0,337522
ST	100,0000	0,411413		
UA 10 μM	102,7712	0,570950	0,949340	0,744263
UA 50 μM	99,20823	1,577582	0,843583	0,901621
Mix **4a/4b** 2 μM	100,0000	1,900238	0,874994	0,874994
Mix **4a/4b** 5 μM	100,3167	1,380491	0,886203	0,863011
Mix **4a/4b** 10 μM	98,89153	1,038391	0,829652	0,910992
Mix **4a/4b** 50 μM	72,05067	13,02039	0,002231	0,999980

post hoc (Tukey)	NST	ST	UA 10 μM	UA 50 μM	Mix **4a/4b** 2 μM	Mix **4a/4b** 5 μM	Mix **4a/4b** 10 μM
NST							
ST	0,872769						
UA 10 μM	0,978886	0,999867					
UA 50 μM	0,821027	1,000000	0,999308				
Mix **4a/4b** 2 μM	0,872769	1,000000	0,999867	1,000000			
Mix **4a/4b** 5 μM	0,890910	1,000000	0,999941	1,000000	1,000000		
Mix **4a/4b** 10 μM	0,798015	1,000000	0,998808	1,000000	1,000000	0,999999	
Mix **4a/4b** 50 μM	0,001169	0,013955	0,006329	0,017489	0,013955	0,012743	0,019138

Table S4. Post hoc analysis of effects of UA and Mix **4a**/**4b** on THP-1 derived macrophage viability measured using NRU assay 24 hours after stimulation. Cells viability presented as a ratio of the experimental group to LPS stimulated cells. ST group. NST – non-stimulated control, ST – LPS stimulated control

	% of PI (-) cells	SEM	post hoc (Dunett) p (<NST)	p (>NST)
NST	81,59444	1,249789		
UA 50 µM	76,06222	1,469574	0,004616	0,999968
Mix **4a/4b** 2 µM	81,98778	1,245794	0,914369	0,788404
Mix **4a/4b** 5 µM	82,03286	1,053212	0,916244	0,784895
Mix **4a/4b** 10 µM	79,30889	1,178412	0,295828	0,996675
Mix **4a/4b** 50 µM	5,827778	0,278487	0,000021	0,999968
Parthenolide 25 µm	60,84000	1,462561	0,000021	0,999968

post hoc (Tukey)	NST	UA 50 µM	Mix **4a/4b** 2 µM	Mix **4a/4b** 5 µM	Mix **4a/4b** 10 µM	Mix **4a/4b** 50 µM
NST						
UA 50 µM	0,027443					
Mix **4a/4b** 2 µM	0,999986	0,014390				
Mix **4a/4b** 5 µM	0,999987	0,041670	1,000000			
Mix **4a/4b** 10 µM	0,819902	0,468627	0,686294	0,783358		
Mix **4a/4b** 50 µM	0,000136	0,000136	0,000136	0,000136	0,000136	
Parthenolide 25 µm	0,000136	0,000136	0,000136	0,000136	0,000136	0,000136

Table S5. Post hoc analysis of effects of UA and Mix **4a**/**4b** on THP-monocyte viability measured using PI flow cytometric assay after 24 hours incubation. ST group. NST – non-stimulated control, ST – LPS stimulated control

			post hoc (Dunett)		post hoc (Tukey)						
	TNF-α secretion (%)	SEM	p (<ST)	p (>ST)	NST	ST	UA 2 µM	UA 10 µM	UA 50 µM	Mix **4a**/**4b** 2 µM	Mix **4a**/**4b** 5 µM
NST	6,990748	1,806716	0,000022	0,999964							
ST	100,0000	1,141368			0,000126						
UA 2 µM	82,29229	6,969327	0,009414	0,999964	0,000126	0,099028					
UA 10 µM	78,42103	2,034191	0,002734	0,999964	0,000126	0,019112	0,998471				
UA 50 µM	21,95156	0,749626	0,000022	0,999964	0,252905	0,000126	0,000126	0,000126			
Mix **4a**/**4b** 2 µM	99,95509	3,715590	0,869749	0,873429	0,000126	1,000000	0,100734	0,019515	0,000126		
Mix **4a**/**4b** 5 µM	64,45318	5,397676	0,000022	0,999964	0,000126	0,000131	0,094194	0,335546	0,000126	0,000131	
Mix **4a**/**4b** 10 µM	108,8595	4,459922	0,997793	0,284701	0,000126	0,840996	0,001580	0,000271	0,000126	0,837519	0,000126

Table S6. Post hoc analysis of effects of UA and Mix **4a**/**4b** on TNF-α secretion in THP-1 derived macrophages 3 hours after stimulation. TNF-α secretion presented as a ratio of the experimental group to LPS stimulated cells. ST group. NST – non-stimulated control, ST – LPS stimulated control

			post hoc (Dunett)		post hoc (Tukey)				
	Il-10 secretion (%)	SEM	p (<ST)	p (>ST)	NST	ST	UA 50 µM	Mix **4a**/**4b** 2 µM	Mix **4a**/**4b** 5 µM
NST	28,78721	3,154767	0,000022	0,999968					
ST	100,0000	3,770853			0,000141				
UA 50 µM	79,03216	15,46492	0,166068	0,999431	0,002041	0,535340			
Mix **4a**/**4b** 2 µM	99,70232	1,429118	0,856240	0,864996	0,043415	1,000000	0,950454		
Mix **4a**/**4b** 5 µM	125,2743	7,250935	0,999872	0,079015	0,000137	0,326567	0,005406	0,885282	
Mix **4a**/**4b** 10 µM	143,6136	8,562598	0,999968	0,001520	0,000137	0,010147	0,000167	0,435253	0,630044

Table S7. Post hoc analysis of effects of UA and Mix **4a**/**4b** on Il-10 secretion in THP-1 derived macrophages 24 hours after stimulation. TNF-α secretion presented as a ratio of the experimental group to LPS stimulated cells. ST group. NST – non-stimulated control, ST – LPS stimulated control

	Il-6 secretion (% of ST)	SEM	post hoc (Dunett) p (<ST)	p (>ST)
NST	0,355629	0,203280	0,000022	0,999970
ST	100,0361	2,667569		
UA 50 µM	25,58801	4,647202	0,000022	0,999970
Mix **4a**/**4b** 2 µM	101,8447	6,602237	0,909063	0,725809
Mix **4a**/**4b** 5 µM	101,0198	3,198856	0,878477	0,778602
Mix **4a**/**4b** 10 µM	91,25331	4,478665	0,217340	0,997386

post hoc (Tukey)	NST	ST	UA 50 µM	Mix **4a**/**4b** 2 µM	Mix **4a**/**4b** 5 µM
NST					
ST	0,000145				
UA 50 µM	0,001181	0,000145			
Mix **4a**/**4b** 2 µM	0,000145	0,999631	0,000145		
Mix **4a**/**4b** 5 µM	0,000145	0,999982	0,000145	0,999992	
Mix **4a**/**4b** 10 µM	0,000145	0,664977	0,000145	0,468793	0,557923

Table S8. Post hoc analysis of effects of UA and Mix **4a**/**4b** on Il-6 secretion in THP-1 derived macrophages 24 hours after stimulation. TNF-α secretion presented as a ratio of the experimental group to LPS stimulated cells. ST group. NST – non-stimulated control, ST – LPS stimulated control
